# Stimuli-Responsive Nanomedicines for the Treatment of Non-cancer Related Inflammatory Diseases

**DOI:** 10.1021/acsnano.5c00700

**Published:** 2025-04-18

**Authors:** Jingjing Yang, Anne des Rieux, Alessio Malfanti

**Affiliations:** † UCLouvain, Louvain Drug Research Institute, Advanced Drug Delivery and Biomaterials, Avenue Mounier 73 B1.73.12, 1200, Brussels, Belgium; ‡ Department of Pharmaceutical and Pharmacological Sciences, University of Padova, Via F. Marzolo 5, 35131 Padova, Italy

**Keywords:** drug delivery, stimuli-responsive, nanomedicine, inflammation, biomaterials, linking chemistry, therapeutic application, inflammatory diseases

## Abstract

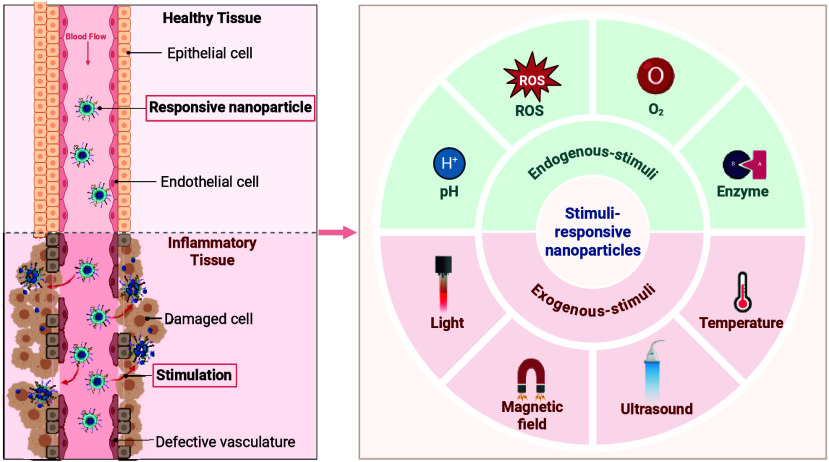

Nanomedicines offer a means to overcome the limitations associated with traditional drug dosage formulations by affording drug protection, enhanced drug bioavailability, and targeted drug delivery to affected sites. Inflamed tissues possess unique microenvironmental characteristics (including excessive reactive oxygen species, low pH levels, and hypoxia) that stimuli-responsive nanoparticles can employ as triggers to support on-demand delivery, enhanced accumulation, controlled release, and activation of anti-inflammatory drugs. Stimuli-responsive nanomedicines respond to physicochemical and pathological factors associated with diseased tissues to improve the specificity of drug delivery, overcome multidrug resistance, ensure accurate diagnosis and precision therapy, and control drug release to improve efficacy and safety. Current stimuli-responsive nanoparticles react to intracellular/microenvironmental stimuli such as pH, redox, hypoxia, or specific enzymes and exogenous stimuli such as temperature, magnetic fields, light, and ultrasound via bioresponsive moieties. This review summarizes the general strategies employed to produce stimuli-responsive nanoparticles tailored for inflammatory diseases and all recent advances, reports their applications in drug delivery, and illustrates the progress made toward clinical translation.

## 1 Introduction

The basic pathological process of inflammation represents a defense mechanism that protects against external/internal stimuli, including pathogens (e.g., microbial and viral infections), irritants (e.g., allergens, radiation, and toxic chemical products), and apoptotic cells (e.g., as a consequence of injuries and autoimmune and chronic diseases).^1^

Acute inflammation represents a short-term response that normally results in healing; however, chronic inflammation represents a prolonged, dysregulated, and maladaptive response and involves active inflammation, tissue destruction, and multiple failed attempts at tissue repair.^2^ Inflammation entails complex events relating to the local vascular and immune systems involving cells such as leukocytes and macrophages within the damaged tissue, which creates a unique microenvironment characterized by elevated levels of cytokines/chemokines and reactive oxygen species (ROS), an acidic pH, and hypoxia.^3^ Chronic inflammation represents a hallmark of many pathologies, including autoimmune disorders (e.g., inflammatory bowel disease [IBD]), inflammatory arthritis (e.g., rheumatoid arthritis and osteoarthritis), neurodegenerative diseases (e.g., Alzheimer’s disease and multiple sclerosis), and cardiovascular diseases (CVDs).^4,5^

The treatment of chronic inflammation generally employs nonsteroidal anti-inflammatory drugs (NSAIDs) or glucocorticoids as major or auxiliary options, which require frequent or continuous treatment;^6,7^ however, currently available treatment strategies suffer from multiple limitations, such as adverse systemic effects, the inability to achieve adequate local drug concentrations, poor drug tissue distribution, gastrointestinal ulcer formation (if orally administered), and difficulties in maintaining stability in circulation. Reformulating drugs as nanomedicines represents an exciting strategy to address these limitations.^8,9^

Stimulus-responsive nanoparticles (SR-NPs) represent an expanding class of nanosized delivery systems designed to promote “on-demand” drug release in response to an intrinsic/extrinsic stimulus. The advantages of SR-NPs reside in precise and controlled drug release after exposure to stimuli specific to the targeted environment; in the case of this review, inflamed tissues.^10,11^ Examples of stimuli-responsive nanomedicine formulations include liposomes,^12^ polymers,^13^ micelles,^14^ dendrimers,^15^ and inorganic nanoparticles,^16^ which have been designed to release active agents in response to internal (e.g., acidic pH, hypoxia, and elevated levels of ROS or specific enzymes) and/or external stimuli (e.g., heat, magnetic fields, light, or ultrasound).^17,18^

This review summarizes recent progress in designing and evaluating SR-NPs for therapeutic applications in a pathological and inflammatory context.

## 2 Inflammation

Inflammation represents an adaptive response to noxious conditions that begins with an initial response of the body to harmful stimuli. In most cases, acute inflammatory responses remain limited to a prescribed area and result from the accumulation of leukocytes in the affected tissues, which act to remove debris and dead cells to promote subsequent repair.

In response to a foreign body (e.g., bacteria), pro-inflammatory cells such as M1-like macrophages and polymorphonuclear neutrophils (PMNs) accumulate in inflamed tissue, surround foreign bodies, and form phagosomes to remove the “bad actor”.^19^ During the clearing process, elevated levels of cytotoxic effectors such as ROS,^19,20^ tumor necrosis factor (TNF-α), interleukin (IL)-1β, IL-6, and myeloperoxidase accumulate in inflamed tissues.^21,22^ If inflammation goes unresolved and becomes chronic (lasting for weeks, months, and even years), then fibrosis and dysfunction can spread to the detriment of surrounding tissues and organs. Persistence of chronicity occurs when the inflammatory response becomes exaggerated when compared to the extent of the danger initially targeted for elimination.^23^

Unresolved inflammation represents a pivotal factor in developing a wide variety of diseases, such as cancer,^2,8,24^ vascular inflammation, arthritis, IBD, and neurodegenerative diseases.^25−28^ The mechanisms underpinning chronic inflammatory diseases remain incompletely elucidated but involve autoimmune reactions, infections, metabolic disorders, and genetics (among other factors).^29,30^

**Figure 1:**
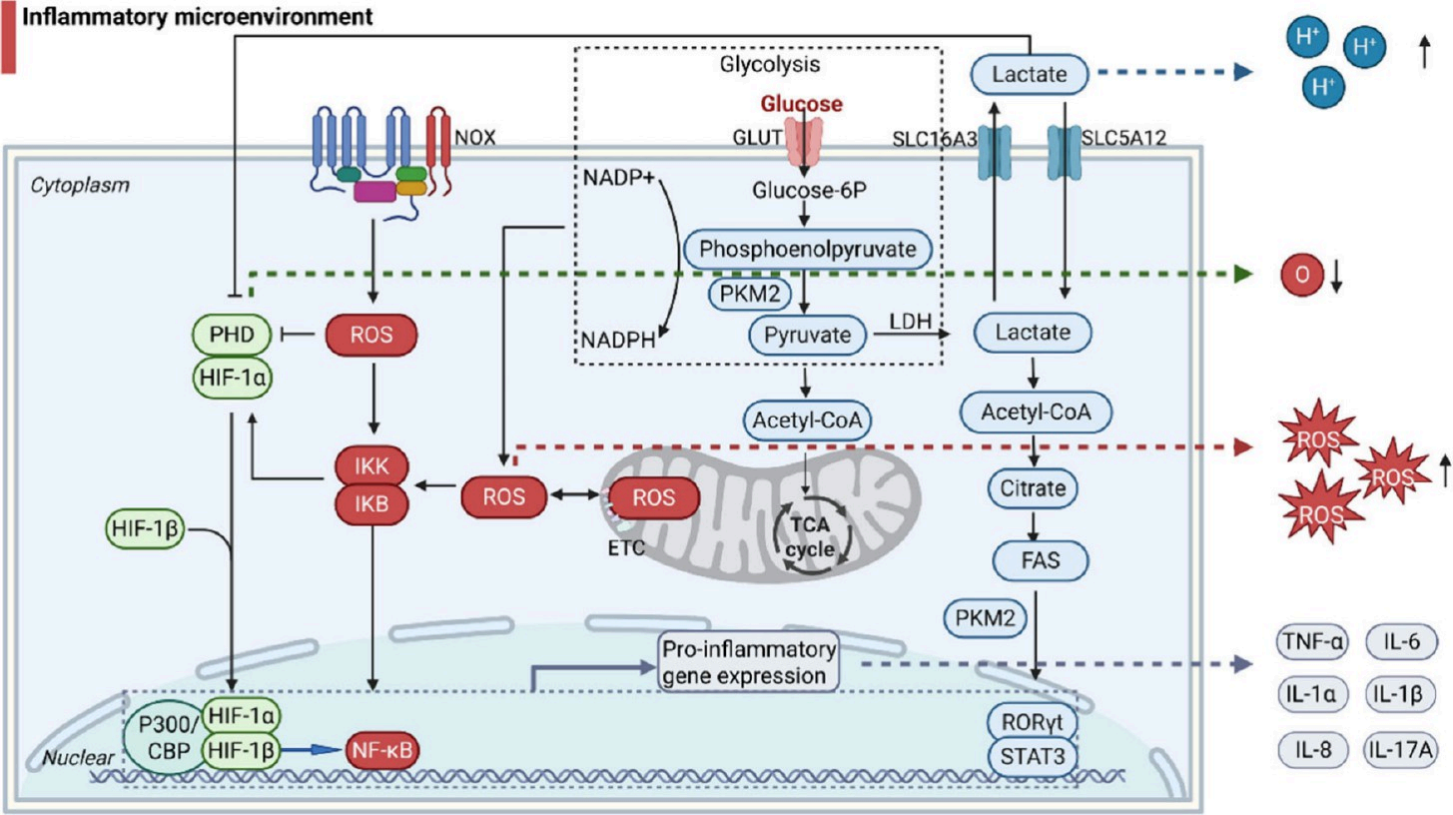
Interactions between pH, reactive oxygen species, and hypoxia in inflammation. Glycolytic metabolism produces lactate under hypoxic conditions, and lactate uptake cells increase citrate and acetyl-CoA levels and initiate an anabolic response leading to fatty acid synthesis (FAS) which promotes the translocation of pyruvate kinase M2 (PKM2) in the nucleus, the activation of downstream retinoic acid-receptor-related orphan nuclear receptor γt (RORγt) and the phosphorylation of signal transducer and activator of transcription 3 (STAT3), with both mechanisms contributing to pro-inflammatory cytokine production. Elevated levels of reactive oxygen species (ROS) activate the NF-κB pathway via oxidation and activation of the inhibitor of NF-κB (IκB) kinases (IKK). ROS production and lactate accumulation are linked to hypoxia through the HIF-1α pathway. Under hypoxic conditions, PHD1–3 activity is inhibited, allowing HIF-1α subunits to translocate to the nucleus, dimerize with HIF-1β and its cofactor p300/CBP, and bind to NF- κB DNA motifs, leading to the release of pro-inflammatory cytokines (TNF-α, IL-6, IL-1α, IL-1β, IL-8 and IL-17A). Created with BioRender.com.

Consequently, a deeper understanding of the pro-inflammatory microenvironment remains crucial to developing strategies to detect and treat inflammation-related diseases. Four elements of the inflammatory environment represent areas of interest from a drug delivery point of view to SR-NP development: (i) acidic pH, (ii) high ROS levels, (iii) hypoxia, and vi) the cleavage activity of specifically upregulated enzymes (Figure 1).

### 2.1 Markers of Inflammation: Cytokines

Cytokines are small proteins (molecular weight <40 kDa) that have an important role in inflammation and its regulation. They are produced by cells to regulate and influence the immune response after injuries. The release of pro-inflammatory cytokines leads to the activation of immune cells and production and the release of more cytokines. A detailed description of the role of cytokines in inflammation has been described^31^ before and falls outside of the scope of this review. Nonetheless, we will briefly discuss the key cytokines involved in noncancer-related diseases that are relevant to this review.

- *TNF-α* is a type II transmembrane protein that can be cleaved by a disintegrin and metalloproteinase (ADAM)-17 into its soluble form, thereby enhancing its biological activity.^32,33^ TNF-α is produced by macrophages and T cells, as well as B cells, neutrophils, and endothelial cells;^34^
- *IL-6* is implicated in autoimmune diseases, bacterial infections, and metabolic side effects.^35^ From a structural point of view, IL-6 is composed of four α-helices and is secreted by T-cells, monocytes, endothelial cells, and fibroblasts.^36^ Importantly, IL-6 affects adaptative immunity by promoting CD4+ T cells.
- *IL-1α and IL-1β* were the first cytokines to be discovered in 1974 by Charles A. Dinarello.^37^ Although IL-1α and IL-1β are encoded by different genes, they can bind the same IL-1 receptor (IL-1R), although IL-1α has more affinity for the subtype 1 (IL-1R1) while IL-1β has more affinity for the subtype 2 (IL-1R2).^38^ IL-1β is secreted by monocytes, macrophages (e.g., microglia or Kupffer cells), and dendritic cells after activation of the pattern recognition receptors (PRR) by pathogen-associated molecular patterns (PAMP) or damage-associated molecular patterns (DAMP).^39^ IL-1α is mainly produced by activated macrophages, as well as neutrophils, epithelial cells, and endothelial cells.^40^
- *IL-8* was first identified as a chemoattractant for neutrophils.^41^ It is produced by macrophages and smooth muscle cells while endothelial cells can accumulate IL-8 in vesicles known as Weibel–Palade bodies.^42,43^
- *IL-17A* is a pro-inflammatory cytokine that is mainly produced by Th17 cells but also by neutrophils, CD8+ T cells, and natural killer (NK) cells and is involved in mediating pro-inflammatory responses by triggering the production of many other cytokines (e.g., IL-1β, IL-6, and TNF-α) and ensures crosstalk between lymphocytes and phagocytes.^44^

### 2.2 Extra- and Intracellular Acidosis Are Signals of Inflammation

pH values in the cytoplasm, blood, and normal tissues lie at around 7.4 but decrease to between 6 and 4 in endosomal/lysosomal organelles.^45^ Deviations from normal tissue pH have been linked to various pathological conditions, such as ischemia/reperfusion, infection, wounds, autoimmune diseases (e.g., multiple sclerosis), and local/systemic inflammatory disorders (e.g., sepsis and IBD).^46,47^ For example, the pH of peritoneal fluid in patients suffering from intra-abdominal infection can decrease from 7.5–8 to below 7.1, while the joint fluid of rheumatoid arthritis patients can reach a value of 6.0.^47^

Under inflammatory conditions, elevated hypoxia, the inactivation of the pentose phosphate pathway (PPP) and tricarboxylic acid cycle (TCA cycle), and increased glycolysis prompt lactate accumulation and acidosis at the affected site.^48^ Lactate exerts immunomodulatory effects after absorption by infiltrating macrophages, producing increased lactate levels in response to hypoxia and inflammatory activation.^49^ In inflamed sites, lactate amplifies inflammation and prompts the entrapment of T cells and the production of pro-inflammatory cytokines.^50^

### 2.3 Reactive Oxygen Species Promote Endothelial Dysfunction and Tissue Injury

Chronic or prolonged ROS production remains central to inflammatory disease progression.^51,52^ ROS, including peroxides such as H_2_O_2_ and superoxides such as O^2·–^, represent essential signaling molecules that regulate metabolism and inflammatory responses.^52^ Transmembrane nicotinamide adenine dinucleotide phosphate (NADPH) oxidases (NOXs) and the mitochondrial electron transport chain (ETC) represent the primary endogenous enzymatic sources of O^2·–^ and H_2_O_2_.^51^ NOX functions from within specialized redox-active endosomes that form in response to specific extracellular stimuli and allow H_2_O_2_ compartmentalization for local redox-mediated regulation.^53^ In addition to intracellular sources, oxidant production also occurs due to environmental exposures and the accumulation of physical/psychological stress.^54^ Lipid-derived ROS production involves the oxidation of polyunsaturated fatty acids and the formation of lipid hydroperoxides and related peroxyl and alkoxyl radicals, which affect redox signaling (especially immune signaling).^55−57^

ROS act as signaling molecules and inflammatory mediators; however, they become deleterious to cells at high concentrations as they oxidize protein and lipid cellular constituents and damage DNA.^51^ ROS can open interendothelial junctions, promote the migration of inflammatory cells across the endothelial barrier, and cause tissue damage.^20,58^ ROS also activate many inflammatory pathways to regulate inflammation (e.g., the nuclear factor kappa B (NF-κB) pathway), hypoxia-inducible factor (HIF) and the response to hypoxia, glyceraldehyde 3-phosphate dehydrogenase (GAPDH) dehydrogenase, and metabolic adaptation.

### 2.4 Hypoxia Environment Induces Immune Cell Dysregulation

Hypoxia occurs under many pathological environments, including those associated with tumors, infections, ischemia, and inflammation.^59^ Pathological hypoxia drives tissue dysfunction and disease development through immune cell dysregulation.^60^ Levels of HIF - the master regulator of oxygen homeostasis that functions by initiating cellular responses to altered oxygen levels^61^ - quickly increase under hypoxic conditions. Tissue oxygenation regulates the expression of the HIF-1α subunit of HIF-1, while HIF-1β is constitutively expressed.^62^ HIF-1α also represents a significant metabolic regulator of inflammation and infection.^63^

### 2.5 Alteration of Enzymatic Cleavage Functionality

The structural characteristics, regulation, and mechanisms of action of certain enzymes become altered in specific pathological states.^64^ Enzymes such as cyclooxygenase (COX) and human Jumonji C domain-containing (JMJD) proteins play critical roles in the development of inflammation. COX proteins promote the production of prostaglandins, which represent critical mediators of the inflammatory cascade.^65^ COX inhibitors (such as NSAIDs) have been widely used in clinics to decrease inflammation. JMJD proteins function as epigenetic modulators in physiological and pathological processes through their histone lysine and arginine demethylase activities; however, JMJD2A promotes the transactivation of pro-inflammatory cytokines,^66^ and blocking JMJD activity can attenuate acute kidney injury associated inflammation.^67^

Members of the matrix-metalloproteinase (MMP) family degrade or cleave components of the extracellular matrix (ECM) and also have significant implications in inflammatory processes.^68^ MMPs are a class of proinflammatory markers that belong to a zinc-dependent endopeptidase family and relative subfamilies (such as gelatinases, collagenases, and stromelysins). The MMP expression is increased by proinflammatory cytokines. The main MMP subtypes, MMP-2 and MMP-9, are upregulated in inflamed tissues during chronic inflammatory conditions such as obesity, arthritis, and atherosclerosis.^69^ Additionally, MMP-13 levels are elevated in certain inflamed tissues, particularly in osteoarthritis.^70,71^ MMP expression levels increased in a lipopolysaccharide-(LPS) induced model of corneal inflammation, although MMP blockade inhibited LPS-induced inflammation.^72^ Additionally, the nucleoside triphosphate diphosphohydrolase (NTPDase) family contributes to and controls the pathophysiology of infectious and inflammatory diseases and injury;^73^ as such, they are employed in therapeutic approaches in certain immune diseases and inflammation.^74^

Cathepsins are a subgroup of proteases organized into three main classes: cysteine, aspartic, and serine proteases. This family includes several subtypes, such as cathepsins B, D, K, L, S, and C, each exhibiting unique expression patterns and biological functions. Cathepsins are primarily located in lysosomes, where they function in protein degradation. Emerging research highlights their crucial role in immune-related diseases, suggesting their potential as therapeutic targets.^75^

## 3 Stimuli-Responsive Nanoparticles: Rationale and Mechanism

As a drug delivery tool, nanoparticles aim to maximize the therapeutic efficacy of a given drug by transporting and releasing the drug to a target site (using passive or active targeting) while minimizing off-target accumulation.^76^ Biocompatible drug delivery systems can be engineered with a wide variety of functions in mind—including enhanced pharmacological activity and pharmacokinetic properties, reduced drug toxicity, accurate active targeting of desired sites, and controlled release - as a response to microenvironmental stimuli.^77^ The formulation of drugs/active molecules as nanomedicines has been successfully translated to the clinic;^78,79^ examples included the lipid nanoparticle employed to deliver the Coronavirus disease 2019 mRNA vaccine.^80^

Among the large arsenal of nanoparticles employed in drug delivery, SR-NPs have quickly become a valuable and widely employed precision tool.^13^ SR-NPs have already been preclinically applied in treating inflammatory diseases such as IBD, rheumatoid arthritis, and CVDs;^81,82^ furthermore, SR-NPs exhibit additional advantages compared to conventional nanomedicines, including enhanced control over the location and timing of drug delivery, resulting in higher therapeutic efficiency and reduced side effects.^83^

### 3.1 Stimuli-Responsive Nanoparticles and Endogenous Stimuli

**Figure 2:**
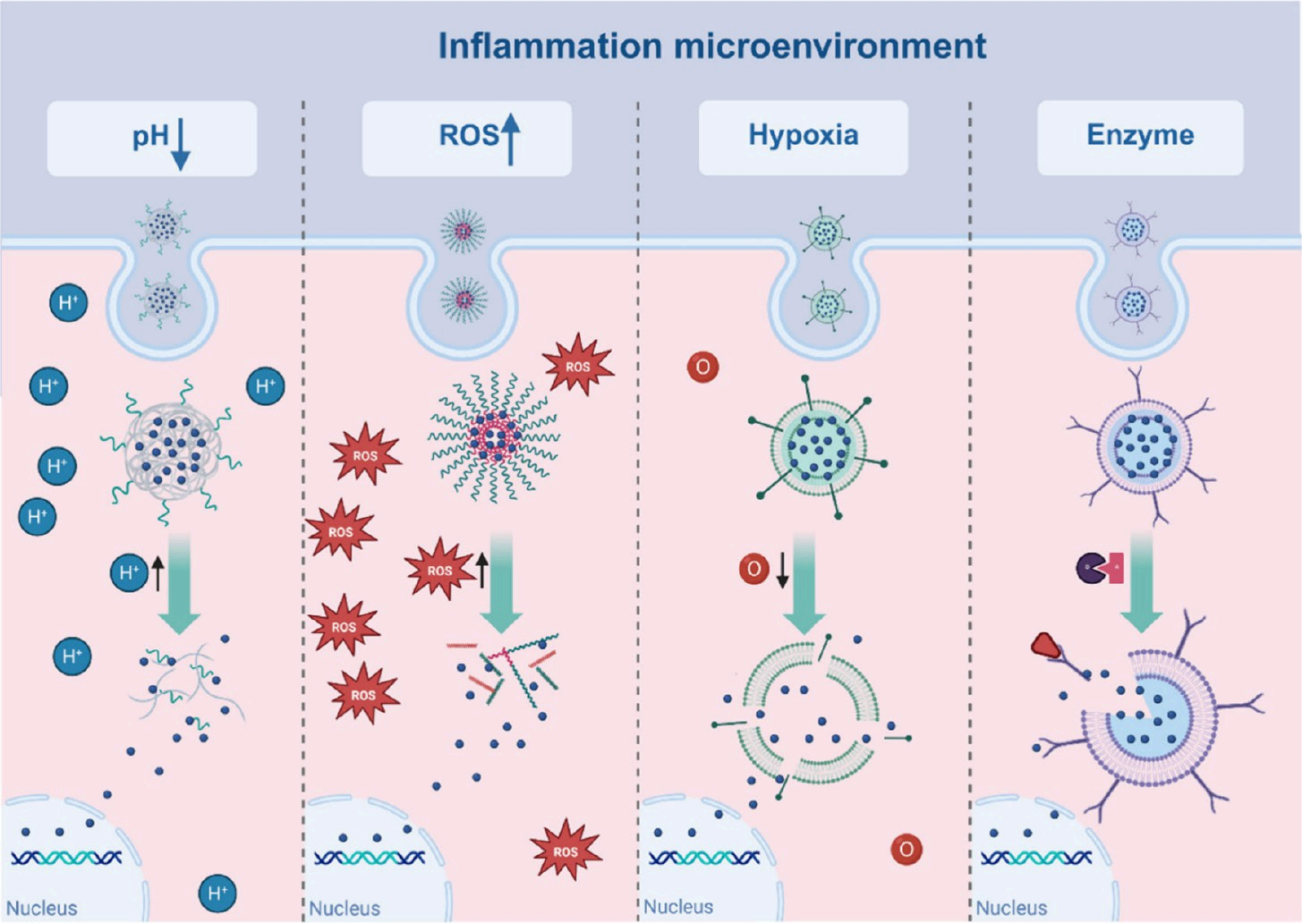
Stimuli-responsive nanoparticle drug delivery systems triggered by endogenous stimuli. Stimuli-responsive nanoparticles encountering endogenous stimuli induced by inflammatory diseases (e.g., low pH, high ROS, low oxygen, and changes in enzyme levels) support targeted drug release at inflammatory sites. Created with biorender.com.

The altered microenvironment associated with acute/chronic inflammation can be leveraged to trigger localized drug release by engineering nanomedicines via bioresponsive linking moieties.^84^Figure 2 provides a schematic representation of various endogenous stimuli-based multifunctional nanocarriers and applications, while Table 1 summarizes those SR-NPs triggered by endogenous stimuli and their application.

**Table 1:**
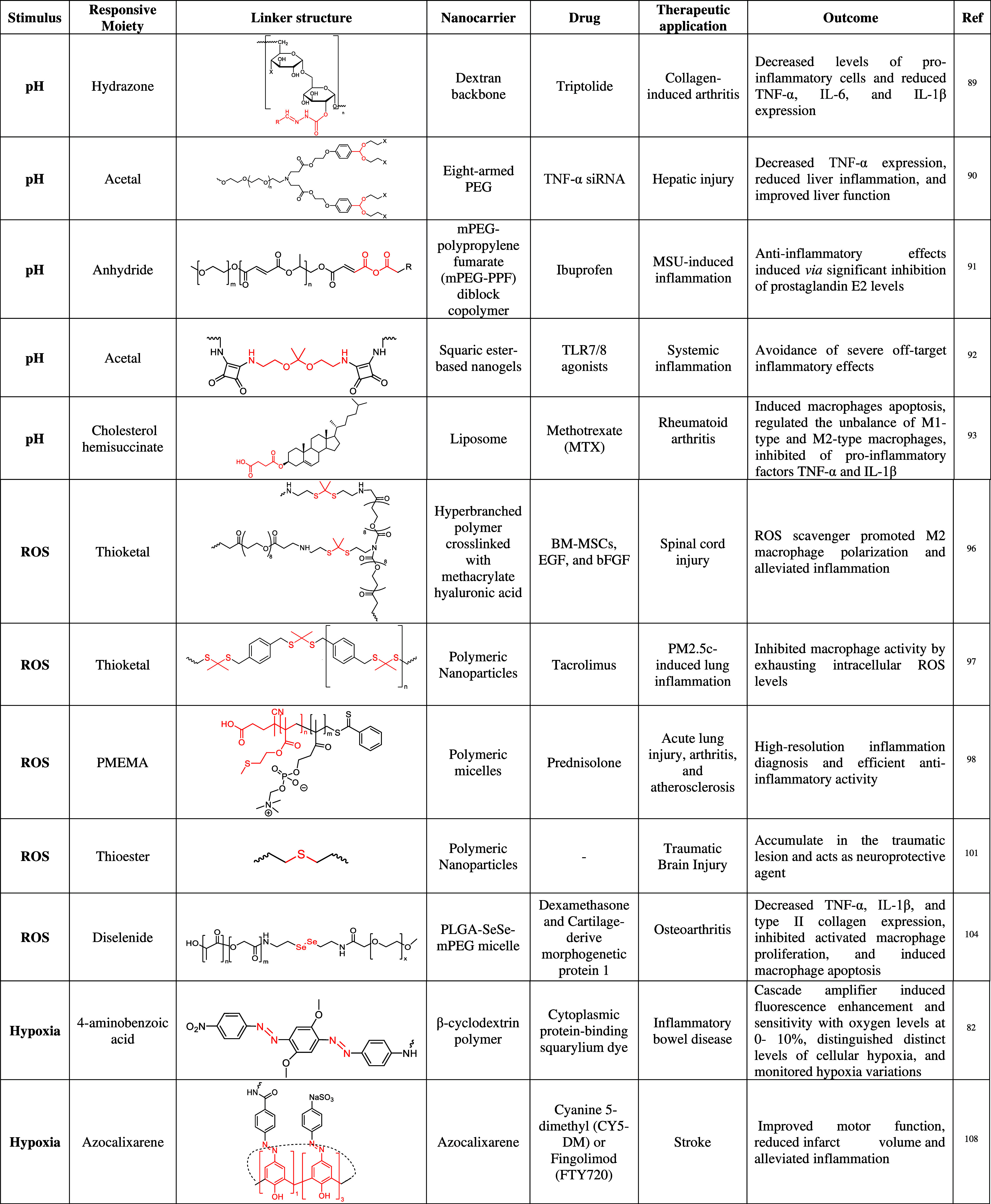

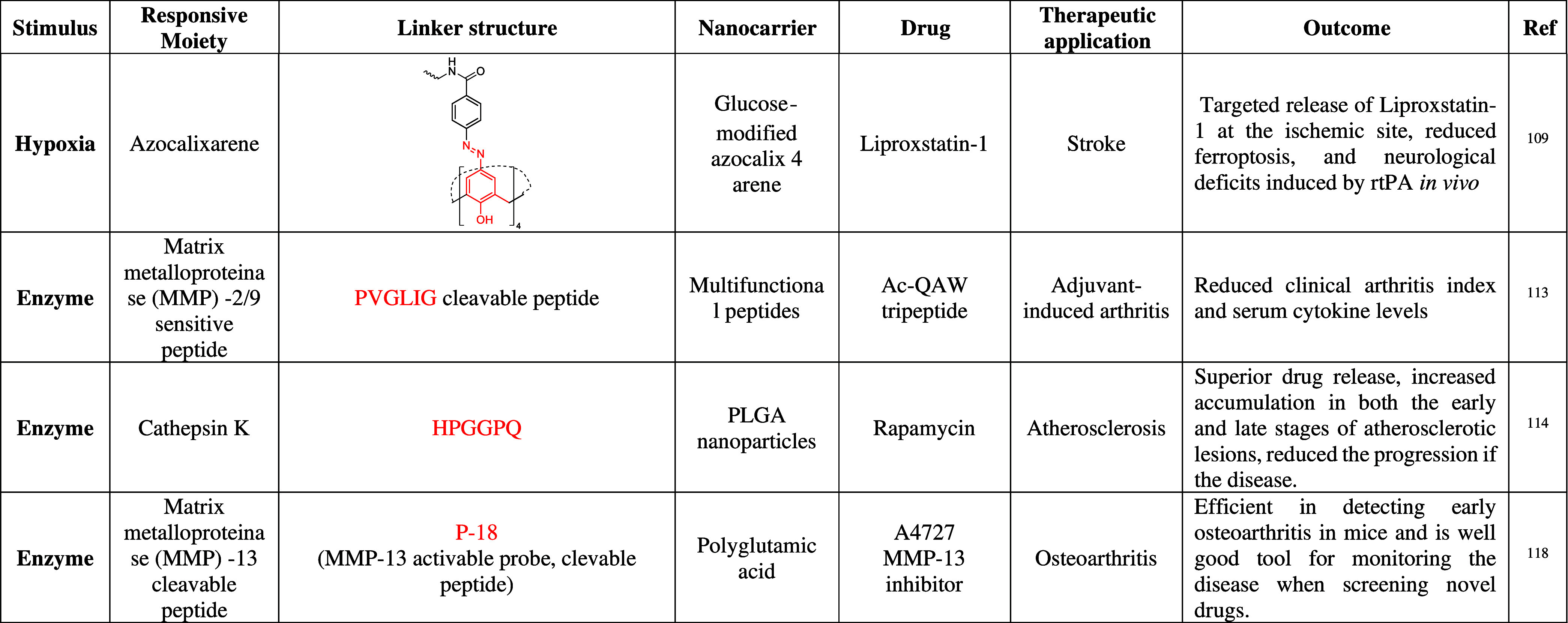
Nonexhaustive Summary of Stimuli-Responsive Nanoparticles Triggered by Endogenous Stimuli with a Focus on the Linking Chemistry Adopted^a^

a The linker that triggers the release of the cargo has been highlighted in red.

#### 3.1.1 pH-Responsive Nanoparticles

pH-responsive nanoparticles display stability at physiological pH levels (around 7.4) but undergo alterations of their material structure/surface characteristics and release drugs when encountering pH values of 5.5–6.5 that characterize sites of inflammation.^85,86^

The two main strategies to design pH-responsive nanoparticles for therapeutic delivery involve (1) pH-labile bioresponsive linkers that allow the nanoparticle to disassemble at defined pH values such as hydrazone, acetal/ketal, boronic acids, imine, ortho ester, cis-aconityl group, and β-thiopropionate moieties^85,87^ or (2) charge-shifting polymers.^87,88^

**Figure 3:**
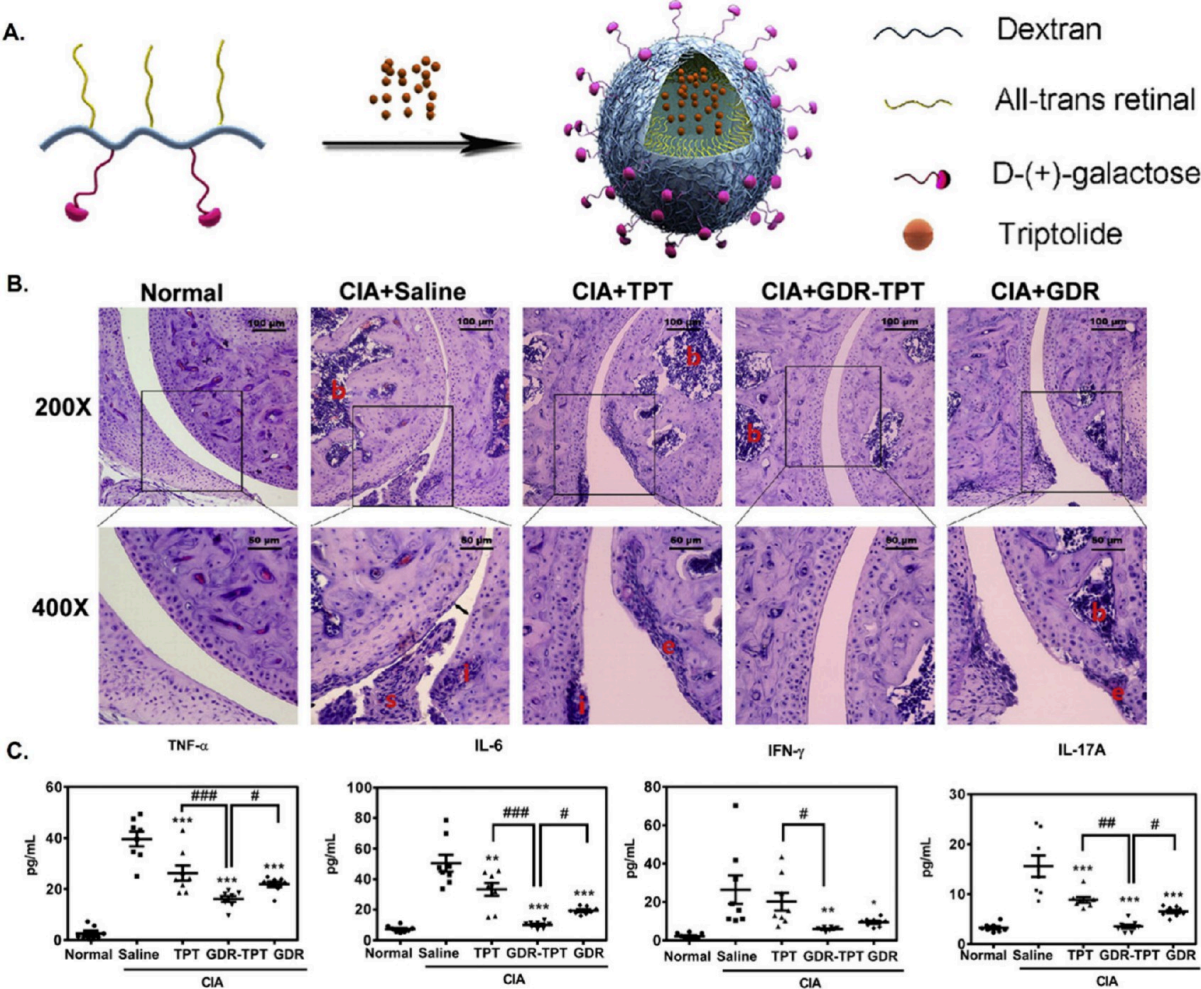
GDR-TPT: An inflammation-targeted pH-sensitive nanoparticle used to treat collagen-induced arthritis. (A) pH-sensitive galactosyl dextran-retinal (GDR) nanoparticles promote intracellular triptolide (TPT) release and induce anti-inflammatory properties. (B) Histologic results of representative joints are shown for each group of mice. Further magnification of the black-bordered box (top) shows typical inflammatory injuries (bottom). Inflammatory infiltration (i), bone destruction (b), and cartilage erosion (e), synovial hyperplasia (s) and narrow joint space (black line). (C) Serum levels of TNF-α, IL-6, IFN-γ, and IL-17A determined by mouse CBA Th1/Th2/Th17 cytokine kit. Reproduced with permission from ref (89). Copyright 2020 Elsevier BV.

Li et al. developed pH-sensitive galactosyl dextran-retinal (GDR) nanoparticles loaded with the anti-inflammatory drug triptolide (TPT) for the treatment of collagen-induced arthritis (CIA).^89^ The authors conjugated all-trans-retinal to a dextran backbone via a pH-responsive hydrazone moiety before grafting with GDR and encapsulating TPT. The intravenous injection of GDR-TPT nanoparticles into CIA mice revealed that nanoparticles targeted macrophages in inflamed lesions, where the cleavage of the pH-responsive linker released TPT to inhibit immune cell infiltration and alleviate the destruction/erosion of cartilage. Specific TPT release also decreased the infiltration of CD3^+^ T cells and F4/80^+^ macrophages, inhibited T helper (Th)1 and Th17 responses, and reduced TNF-α, IL-6, and IL-1β expression (Figure 3). Tang et al. reported the design of pH-responsive nanoparticles that delivered anti-TNF-α small interfering (si)RNAs chemically cross-linked to a multiarmed poly(ethylene glycol) (PEG) nanocarrier via an acid-labile acetal linker to macrophages in a mouse model of inflammation-induced liver damage.^90^ The authors discovered that the selective accumulation of this nanoparticle in the liver induced a 2-fold decrease of TNF-α expression in macrophages compared to “free” TNF-α siRNA following hepatic injury and protected mice from liver damage.

The implementation of charge-shifting polymers represents another approach to integrate pH-responsivity, where acidic pH prompts polymer protonation to induce a charge shift, resulting in nanocarrier disassembly and on-demand drug release. Applying this strategy to pH-responsive nanoparticles requires the introduction of specific functional moieties, such as carboxylic groups or titratable amines.^85^

Seetharaman et al.^91^ conjugated the anti-inflammatory drug ibuprofen to a carboxyl-terminated methoxy polyethylene glycol-polypropylene fumarate (mPEG–PPF) charge-shifting diblock copolymer via anhydride linkages to form amphiphilic polymer-drug conjugates (PDCs) for the treatment of monosodium urate induced inflammation *in vitro*. Introducing the pH-responsive linker N, N′-dimethyl aminoethyl methacrylate (DMAEMA) to cross-link mPEG–PPF endowed pH-responsive characteristics to this PDC. The gradual protonation of the tertiary amine group in DMAEMA upon decreasing pH prompted PDC disruption and destabilization and subsequent ibuprofen release, with the PDC displaying better anti-inflammatory effects than free ibuprofen. In another example, Cai et al.^88^ employed methacrylic acid-methyl methacrylate copolymer (Eudragit S100) and hyaluronic acid (HA) as pH-responsive groups. They adsorbed ES100 and HA onto the surface of chitosan (CS) nanoparticles loaded with tacrolimus (FK506)/HP-β-cyclodextrin (β-CD) through electrostatic interactions to obtain pH-responsive nanoparticles (FK506@EHCh) that they evaluated in a mouse model of IBD. FK506@EHCh nanoparticles displayed pH-responsive characteristics and facilitated an increased concentration of drugs at sites of intestinal inflammation, where they significantly inhibited the inflammatory response and suppressed TNF-α, IL-1β, and IL-6 expression. These results were similar to those of the control group (healthy mice).

Amine groups have also been applied as pH-responsive groups in charge-shifting polymers. Huppertsberg et al.^92^ designed pH-responsive nanogels as versatile nanocarriers to safely deliver TLR7/8-stimulating imidazoquinolines by intravenous administration. They first polymerized a primary amine-reactive methacrylamide monomer bearing a pendant squaric ester amide under controlled reversible addition–fragmentation chain transfer polymerization conditions. Resultant PEG-derived squaric ester amide block copolymers self-assembled into precursor micelles in polar protic solvents, which permitted the encapsulation of a TLR7/8 small-molecule agonist. While they displayed stability at pH 7.4, the nanogels hydrolyzed at pH 5, which shifted nanogel behavior from hydrophobic to hydrophilic via the acid-sensitive cross-linking and prompted core transformation. Encouragingly, this approach reduced the viability of activated macrophages *in vitro* and had an immunomodulatory effect on the systemic inflammation.

Zheng et al.^93^ designed pH-responsive liposomes for delivering drugs specifically to inflamed joints in acidic environments. These liposomes were composed of pH-responsive cholesterol hemisuccinate (CHEMS) and coated with nanoparticles made of methotrexate (MTX)-human serum albumin (HSA) complex (MTX-HSA) forming a drug delivery system called Lipo/MTX-HSA. After intravenous administration, Lipo/MTX-HSA accumulated in arthritic joints and released MTX-HSA, due to the acidic pH (approximately pH 5.5), which reduced the number of fibroblast-synoviocytes and macrophages, alleviating joint inflammation and repair bone erosion in a rat model of rheumatoid arthritis.

pH levels and gradients can be characterized by different organs, tissues, and subcellular compartments and their pathophysiological states. For example, due to pH varying at different locations in the gastrointestinal tract, pH-responsive nanoparticles can be used for oral drug delivery to improve systemic exposure from greater gastric retention, transepithelial transport, and cellular targeting with surface-functionalized ligands. Drawbacks in this strategy include the need for linking chemistries stable at pH 7 that undergo cleavage at pH 6 and the fact that cargo release in the acidic pH of the lysosome can induce unwanted degradation.^87^

#### 3.1.2 ROS-Responsive Nanoparticles

The cellular microenvironment of inflammatory diseases is often characterized by elevated levels of ROS, making redox-responsive nanoparticles - primarily generated via the implementation of tioketal (TK) cross-linkers^94^ - an exciting treatment approach. Li et al.^95^ explored tannic acid (TA)-capped hafnium disulfide (HfS2@TA) nanosheets, a 2D atomic crystal of hafnium-based materials prepared by liquid-phase exfoliation, as high-performance anti-inflammatory nanoagents for the targeted therapy of IBD by oral (40 mg/kg) or intravenous (10 mg/kg) administration. Benefiting from the transformation of the S^2–^/S^6+^ valence state and huge specific surface area, the HfS2@TA nanosheets effectively eliminated ROS/reactive nitrogen species and downregulated the expression of pro-inflammatory factors (e.g., TNF-α, IL-1β, and IL-6) by 35%.

**Figure 4:**
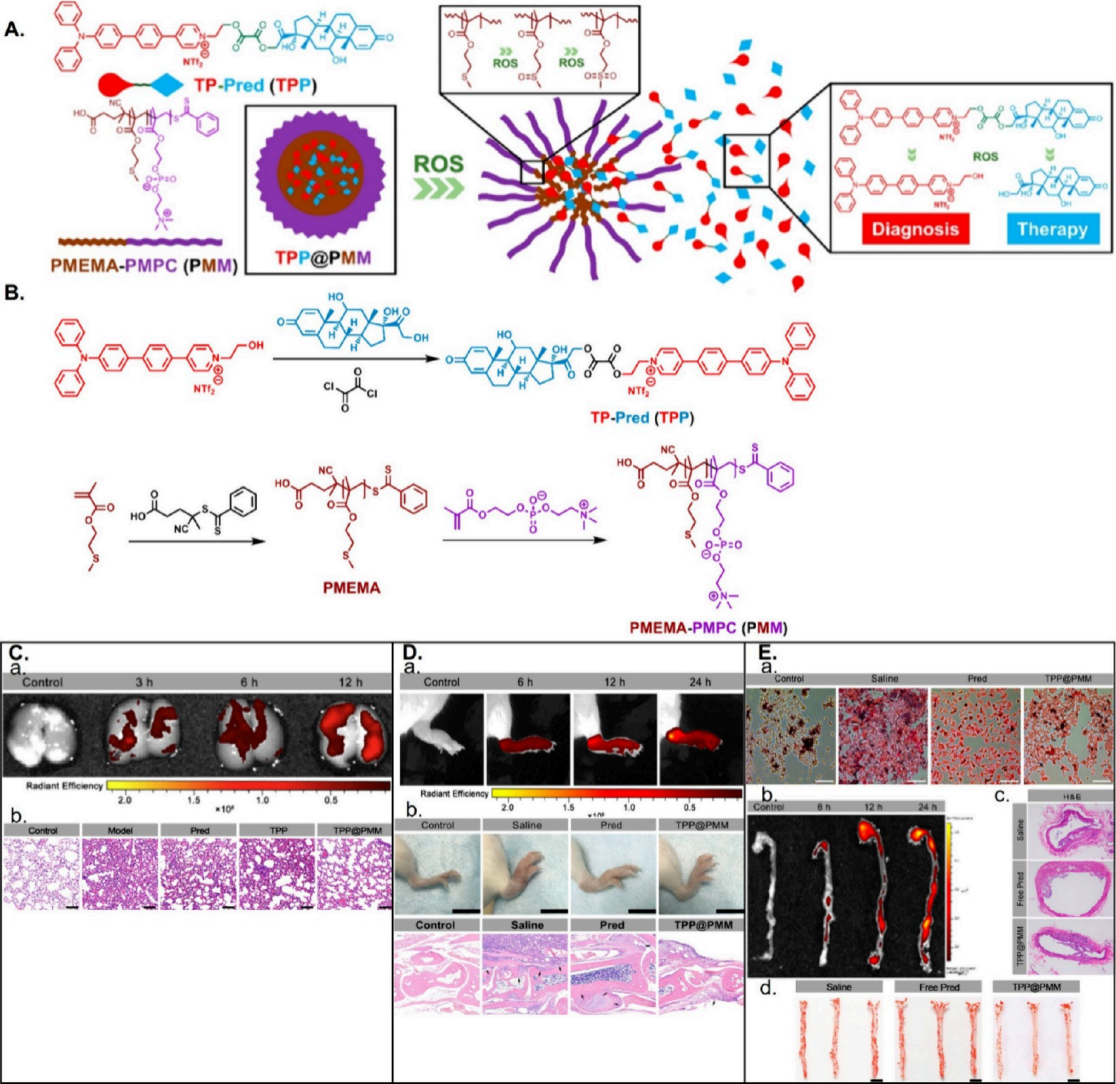
TPP@PMM: a nanoplatform with two-photon imaging and serial redox-responsivity for diagnosing and treating acute lung injury, arthritis, and atherosclerosis. (A) Structure of TPP@PMM for two-photon imaging and serial redox-responsivity. (B) Synthetic route employed for TPP and PMM (2-methylthio ethanol methacrylate)-poly(2-methacryloyloxyethyl phosphorylcholine)). (C) Ex vivo fluorescent images of (a) lungs and hematoxylin and eosin (H&E)-stained sections of pulmonary tissue (b) from acute lung injury (ALI) mice treated with TPP@PMM. (D) Ex vivo fluorescent images of the (a) joint from arthritic mice, (b) images of the right hind limbs, and (c) H&E staining sections of joint tissue from arthritic mice injected with TPP@PMM. (E) (a) Optical microscopy images of oxidized low-density lipoprotein-induced foam cell formation in macrophages, (b) ex vivo fluorescence images of TPP@PMM accumulation in the aorta, (c) H&E staining sections of the aorta, and (d) representative images of en face oil red O(ORO)-stained aortas from atherosclerotic mice injected with TPP@PMM. Reproduced with permission from ref (98). Copyright 2020 American Chemical Society.

Li et al.^96^ employed a thioketal-containing hyperbranched polymer cross-linked with methacrylate hyaluronic acid to form a ROS-responsive and ROS-scavenging hydrogel; of note, thioketal represents a commonly employed ROS-responsive cross-linker. The authors covalently grafted neural-specific peptides to the hydrogel and encapsulated rat-derived epidermal growth factor (EGF), basic fibroblast growth factor (bFGF), and bone marrow mesenchymal stromal cells (BMSCs). The resultant hydrogel responded to and scavenged ROS, polarized M2-like macrophages, alleviated inflammation, and protected BMSCs against oxidative stress when cotransplanted as a component of spinal cord injury (SCI) treatment *in vivo*. In another study, Zhang et al.^97^ developed poly(1,4-phenleneacetonedimethylene thioketal) (PPADT)-derived ROS-responsive nanoparticles loaded with the immunosuppressant agent tacrolimus. The developed nanoparticles demonstrated robust ROS-responsiveness and underwent degradation in a highly oxidative environment. Released tacrolimus effectively inhibited macrophage activity by exhausting intracellular ROS levels and suppressed inflammation to a greater degree than free tacrolimus *in vitro* and *in vivo*. Studies have also reported the application of redox-sensitive coatings to mesoporous silica nanomaterials, liposomes, or dendrimer-drug conjugates containing thiol-cleavable bonds.^85^ For example, the degradation of amphipathic poly(2-(methylthio) ethanol methacrylate) (PMEMA) by a ROS-triggered hydrophobic-to-hydrophilic conversion supported drug release in response to ROS accumulation.^98,99^ The authors created core–shell structured micelles via the self-assembly of PMEMA-poly(2-methacryloyloxyethyl phosphorylcholine) (PMEMA–PMPC or PMM) and then loaded them with the theranostic compound TPP (prednisolone bridged to a two-photon fluorophore [TP] via a ROS-sensitive linker) to form TPP@PMM. The ROS-triggered hydrophobic-to-hydrophilic conversion of PMEMA interrupted the micellar structure to allow TPP release before the cleavage of the ROS-responsive bond in TPP, which resulted in specific prednisolone delivery. This nanoparticle supported the high-resolution diagnosis of inflammation (by specific accumulation) and efficient anti-inflammatory activity in treating acute lung injury (ALI), arthritis, and atherosclerosis and the avoidance of glucocorticoids’ serious side effects (Figure 4).^98^

Miyata et al.^100^ designed a thiolated poly(ethylene glycol)-poly(l-lysine) (PEG–PLL) block copolymer for gene delivery. Through a thorough investigation of the linking chemistry, conjugation strategy, and linker density on the polymeric backbone, the authors evaluated the crucial role of thiols in enhancing pDNA delivery. They found that an optimal thiolation degree (28%) on PEG–PLL led to efficient intracellular gene delivery, achieving higher transfection efficiency compared to suboptimal or nonthiolated micelles. Convertine and colleagues^101,102^ developed an elegant method to produce ROS-responsive core-cross-linked nanoparticles (NPs) for traumatic brain injury. By using a combination of thiol–ene and thiol–Michael chemistry, they produced NPs with low polydispersivity and a high proportion of thioether units that reduce local levels of ROS. In a controlled cortical impact mouse model of traumatic brain injury, the authors observed a rapid accumulation of these NPs and their ability to be retained in the damaged brain, as visualized through fluorescence imaging. Additionally, the NPs reduced neuroinflammation and the secondary spread of injury, as demonstrated by magnetic resonance imaging and histopathology, and improved functional outcomes, as assessed through behavioral analyses, compared with the controls.

Ma et al.^103^ employed fluorophore-cyclodextrin/prednisolone complexes packaged within PMEMA–PMPC-based nanosized micelles, which become activated by locally elevated levels of ROS and lipids to result in anti-inflammatory activity and lipid removal when employed to inhibit atherosclerosis. Wang et al.^94^ synthesized a hyperbranched ROS-sensitive macromer (poly(β-amino ester) (HB-PBAE) with multiacrylate end groups by employing PEG-diacrylate and cysteamine and using the Michael addition approach. They next employed a straightforward protocol based on dopamine polymerization to generate a polydopamine layer deposited on the tanshinone IIA nanoparticles formed from spontaneous hydrophobic self-assembly. HB-PBAE reacted with thiolate-modified hyaluronic acid to form an in situ hydrogel and reported the resultant hydrogel as ROS-responsive, significantly improving cardiac functions after injection and inhibiting the expression of inflammation factors, such as IL-1β, IL-6, and TNF-α.

Similar nanoparticles have employed differing ROS-responsive components, including the selenium–selenium (-SeSe-) element.^104^ Wu et al. synthesized a ROS-responsive nanoparticle assembled using polylactic acid-glycolic acid (PLGA)-SeSe-mPEG, loaded with dexamethasone (DEX) and chondrogenic differentiation factor cartilage-derived morphogenetic protein-1 (CDMP-1) for the treatment of osteoarthritis.^104^ Exposure to 500 μM H_2_O_2_*in vitro* prompted a DEX release rate higher than 60% and a CDMP-1 release rate of 37.7% due to -SeSe- cleavage. *In vivo*, the elevated ROS levels present in arthritic lesions led to -SeSe- linker rupture and the controlled release of DEX and CDMP-1, which inhibited activated macrophage proliferation and increased apoptosis to induce an overall anti-inflammatory effect.

Lipids as well can be derivatized to produce SR-NP. For instance, Tanaka et al.^105^ designed a self-degradable lipid-like material to promote the collapse of lipid nanoparticles (LNPs) and the release of RNA into the cytoplasm. This lipid is based on oleic acid-scaffolds bearing both a disulfide bond and a phenyl ester linkers; the concentrated hydrophobic thiols that are produced by the cleavage of the disulfide bonds due to the higher intracellular levels of glutathione drive an intraparticle nucleophilic attack to the phenyl ester linker, which results in further degradation of the LNP enhancing thus the transfection efficiency. De Lombaerde et al.^106^ developed a series of ionizable biscarbamate lipids (IBLs) for mRNA LNP delivery. The building blocks of these lipids are (1) a dialkyl chain, (2) a homobifunctional activated carbonate ester linker, and (3) an ionizable headgroup. The screening of these lipids was elegantly performed based on their physicochemical properties, *in vivo* biodistribution, and capacity to elicit an immune response against target antigens. The lead ionizable lipid, S–Ac7-DOG, formed into LNPs demonstrated a good biodistribution profile, with high mRNA expression observed in the draining lymph nodes and spleen while exhibiting high-magnitude antigen-specific CD8+ T cell responses. Although these LNPs have been applied for cancer therapy, we do believe that they could have potential in the noncancer related field too (unpublished work from Prof. Anne des Rieux).

On the other hand, the inflamed tissue includes vascular permeability to leukocytes, macrophages, and other immune cells, which can be used as targets for potential therapies. For example, Marotti and co-workers^107^ aimed to stimulate the physiological secretion of glucagon-like peptide 2, a peptide known for its intestinal growth-promoting effects, designing hybrid lipid hyaluronate (HA)-KPV conjugated nanoparticles loaded with teduglutide for combination therapy in IBD. HA-KPV was conjugated using a disulfide bond, aiming to be released after immune cell uptake. The nanocarriers either induced or did not induce immunosuppression, depending on the presence or absence of the hyaluronan-KPV functionalization, displaying. This approach shows potential as a nanoparticle platform for combined mucosal healing and immunomodulation in the treatment of IBD. We do believe this strategy could be leveraged for future treatments of other noncancer related inflammatory diseases.

#### 3.1.3 Hypoxia-Responsive Nanoparticles

**Figure 5:**
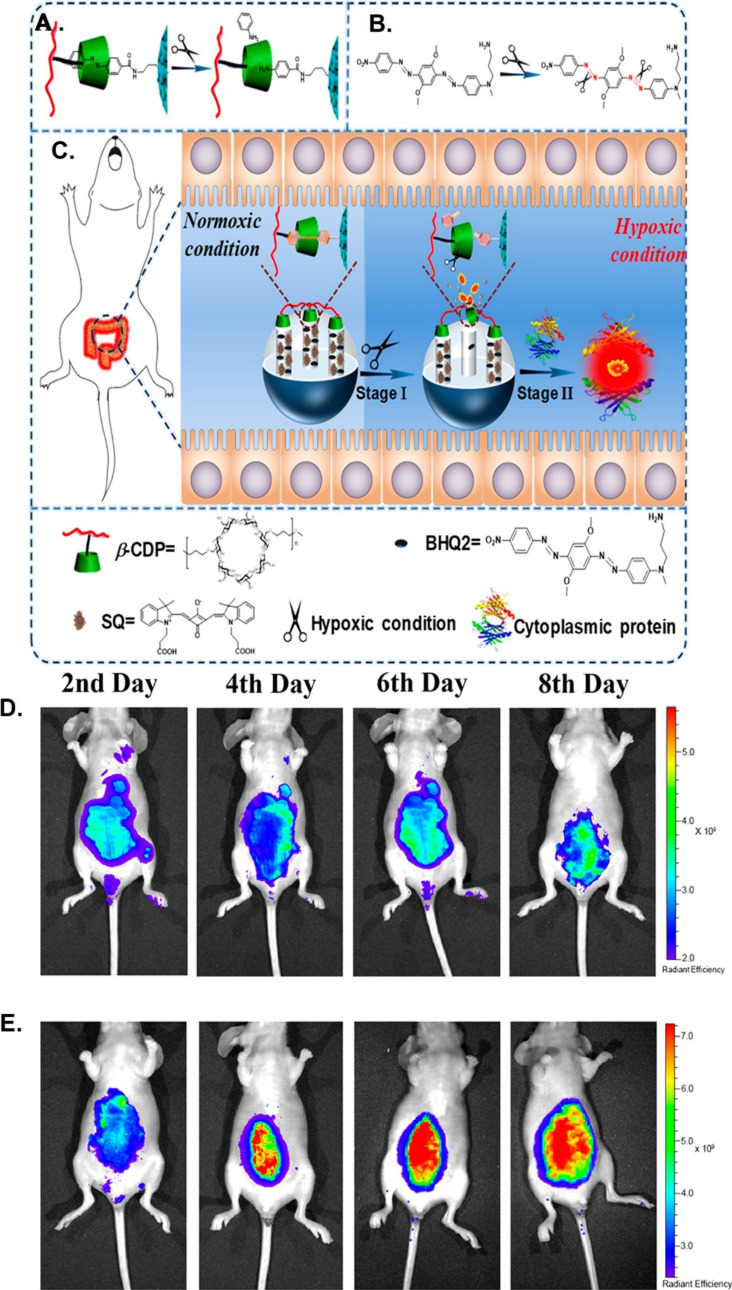
Imaging inflammatory-bowel-disease-associated hypoxia by a hypoxia-activatable and cytoplasmic protein-powered fluorescence cascade amplifier. (A) Hypoxia-responsivity mechanism of azo/β-cyclodextrin polymer (β-CDP) and (B) black hole quencher 2 (BHQ2) response to hypoxia. (C) Imaging of hypoxia associated with inflammatory bowel disease (IBD) by a cascade amplifier based on cytoplasmic protein-powered fluorescence cascade amplification (HCFA). Whole-body fluorescent images (pseudocolor) of nude mice from the (D) control group and (E) acute colitis after intraperitoneal injection of HCFA. Reproduced with permission from ref (82). Copyright 2020 American Chemical Society.

Given the hypoxic nature of the inflammatory tissue-associated microenvironment, some researchers have studied hypoxia-responsive nanoparticles to treat inflammatory diseases. Zhou et al.^82^ took advantage of the reduction of azobenzene to aniline derivatives under hypoxic conditions to create hypoxia-responsive nanoparticles for use in IBD diagnosis. The authors loaded black hole quencher 2 (BHQ2) and cytoplasmic protein-binding squarylium dye (SQ) into a 4-aminobenzoic acid (azo)-modified mesoporous silica nanoparticle (MSN) before combining a β-cyclodextrin polymer (β-CDP) with the azo moiety through the host–guest interaction to form a hypoxia-activatable and cytoplasmic protein-powered fluorescence cascade amplifier (HCFA). The cleavage of the azo bond in response to a hypoxic microenvironment permitted the HCFA to emit fluorescence according to varying oxygen levels, which the authors took advantage of to create an IBD-associated hypoxia detection tool; results showed that the acute colitis region exhibited an intense fluorescence signal (Figure 5).

A recent study investigated a hypoxia-responsive azocalixarene (CA)-Q11 peptide hydrogel designed to suppress inflammation in an ischemic stroke. The hydrogel was loaded with Fingolimod, an FDA-approved drug for the treatment of multiple sclerosis and locally applied in stroke mice model *in vivo*.^108^ Wu et al. used a glucose-modified azocalix[4]arene (GluAC4A) for the targeted delivery to the ischemic site of stroke of liproxstatin-1 (Lip), a ferroptosis inhibitor.^109^ After intravenous injection, GluAC4A nanoparticles loaded with Lip successfully improved drug accumulation in the brain, significantly reducing ferroptosis, blood-brain barrier leakage, and neurological deficits induced by recombinant tissue plasminogen activator (rtPA) in a mouse model of middle cerebral artery occlusion (MCAO).

The development of hypoxia-responsive nanoparticles has supported drug delivery to hypoxic tissues, providing enhanced molecular imaging and treatment by improving drug circulation times and specific drug accumulation;^110^ however, certain problems with hypoxia-responsive nanoparticles remain unaddressed, such as the significant difference in their sensitivity in inflamed regions (O_2_ > 7.6%) compared to, for example, the severe hypoxic state observed in tumors (O_2_ < 1.4%). Hampered by their inadequate sensitivity, it remains a challenge for these probes to be further applied in investigating inflammation-associated hypoxia.^82^

#### 3.1.4 Enzyme-Responsive Nanoparticles

Certain enzymes become overexpressed in diseased/dysregulated tissues, and, as such, we can employ their known substrates as components in a drug delivery system to achieve specific drug release at a target tissue.^17^ Enzyme-responsive nanoparticles release therapeutic drugs via multiple modes, including size shrinkage, surface charge switching, surface ligand activation, and chemical bond cleavage.^111^

**Figure 6:**
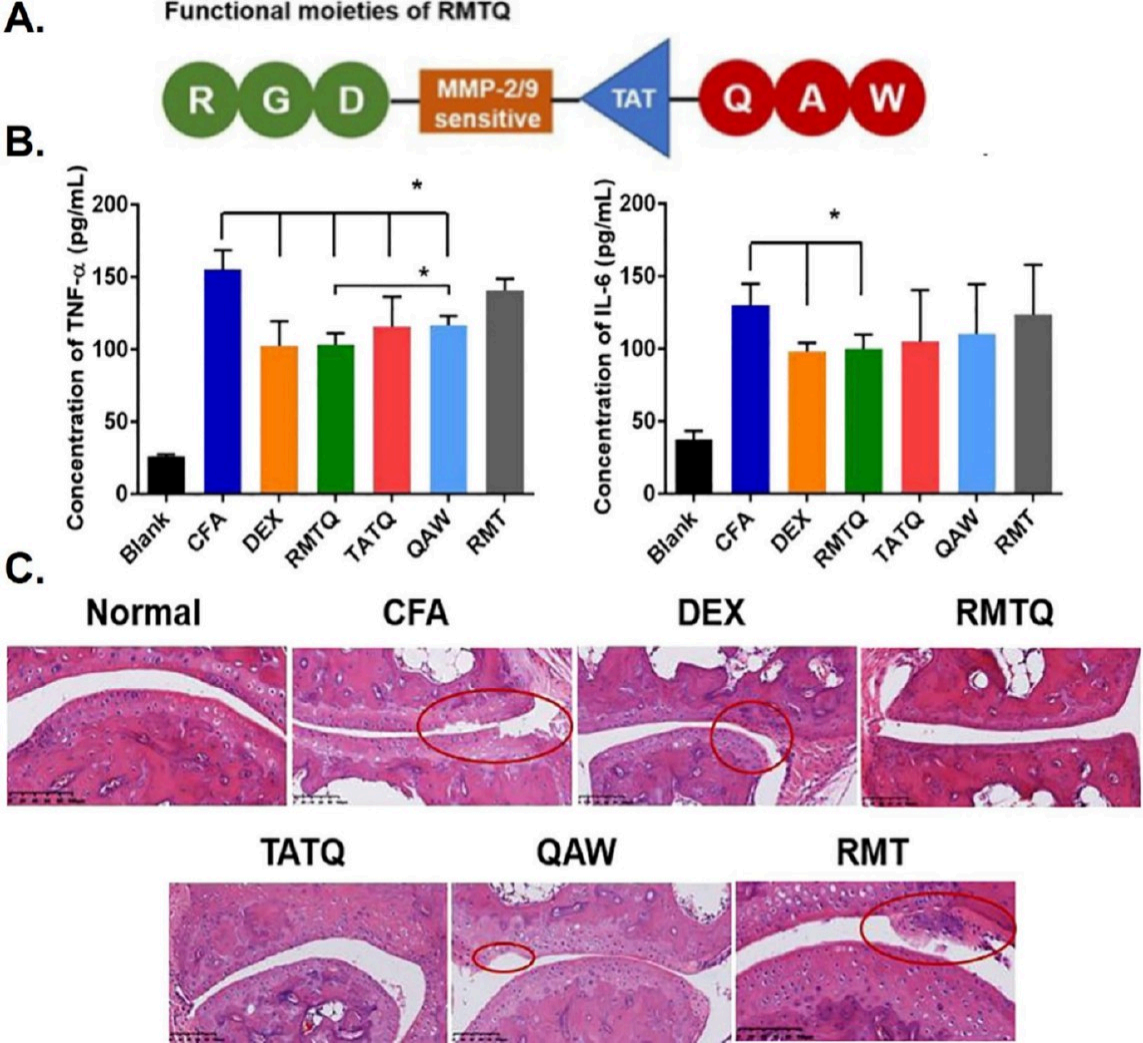
Inhibition of inflammation by RMTQ. (A) Functional moieties of the RGD-MMP-TAT-QAW peptide (RMTQ). (B) Serum concentration of TNF-α and IL-6 in the normal, model group (CFA), Dexamethasone (DEX), RGD-MMP-TAT-QAW (RMTQ), TAT-QAW (TATQ), QAW, and RGD-MMP-TAT (RMT) groups (CFA vs other groups, RMTQ vs QAW). (C) Histological section images of inflamed mice joints after different treatments. Reproduced with permission from ref (113). Copyright 2019 Elsevier BV.

Li et al. employed the acetyl-Gln-Ala-Trp (Ac-QAW) tripeptide obtained from the anti-inflammatory protein Annexin A1, which alleviates inflammation via NF-κB inhibition,^112^ as the starting point for the development of an enzyme-responsive nanoparticle for the treatment of complete Freund’s adjuvant-induced arthritis (AIA) in mice.^113^ They modified Ac-QAW with the cell-penetrating peptide TAT to enhance internalization and then conjugated the arginine-glycine-aspartic acid (RGD) sequence to TAT-QAW using an MMP-2/9 sensitive peptide to improve targeting and ensure responsiveness to inflammation. The RGD-MMP-TAT-QAW peptide (RMTQ) exhibited robust responses to MMP-2/9 and enhanced delivery to the cytoplasm, which prompted a significant reduction in pro-inflammatory TNF-α and IL-6 expression and overall better efficacy than free Ac-QAW in AIA mice, had the same effect as traditional anti-inflammation drug-Dexamethasone (Figure 6).^113^

Based on the fact that atherosclerotic lesions are rich in Cathepsin K, Fang et al. developed PLGA nanoparticles that are responsive to this enzyme for targeted delivery of Rapamycin. The enzyme-responsive component is a small peptide sequence, HPGGPQ. To further enhance nanoparticle accumulation, PLGA was modified with the RGD peptide (RAP@T/R NPs). Cathepsin K facilitated the release of rapamycin, enhancing its release. In contrast, no significant difference was observed with the nonresponsive nanoparticles. RAP@T/R NPs demonstrated prolonged blood retention and increased accumulation in both the early and late stages of atherosclerotic lesions compared to the controls. Importantly, RAP@T/R NPs significantly inhibited the progression of atherosclerosis and reduced both systemic and local inflammation compared to the controls.^114^

In the case of liposomes,^115^ typical enzymes that can be leveraged for controlled drug release can be phospholipases, such as phospholipase A2 (PLA2). Li et al.^116^ produced a liposomal hydrogel by embedding a curcumin-loaded liposome made of egg phosphatidylcholine into a gelatin-chitosan hydrogel. The authors achieved drug release after PLA2 hydrolysis in wound exudate for infection treatment.

Joshi and colleagues^117^ obtained an injectable self-assembly triglycerol monostearate (TG-18) hydrogel loaded with triamcinolone acetonide (TA) for the treatment of inflammatory arthritis. MMPs, which are overexpressed during IA flares, can cleave ester bonds in TG18, leading to TA release. The on-demand sustained release of the drug after intra-articular injection improved the therapeutic efficacy of locally delivered drugs and reduced arthritis severity postinjection.

Polyglutamic Acid (PGA) was conjugated with imaging probes using an MMP-13 cleavable linker and has been exploited as a polymer-probe (P18) to detect early osteoarthritis in mice as well as a tool to monitor the disease when screening novel drugs.^118^

In the case of inflammatory microenvironment of specific enzyme expression, enzyme-responsive nanoparticles support gradual drug release profiles and prolonged therapeutic effects on inflammation,^85^ such as more enzymes at the targeted site and more drugs being released from nanoparticles to treat the infection.^119^ Unfortunately, certain enzymes display disease-specific alterations in expression; therefore, enzyme-responsive nanoparticles are a limited field of application and have not been adapted for widespread use.

### 3.2 Stimuli-Responsive Nanoparticles and Exogenous Stimuli

**Figure 7:**
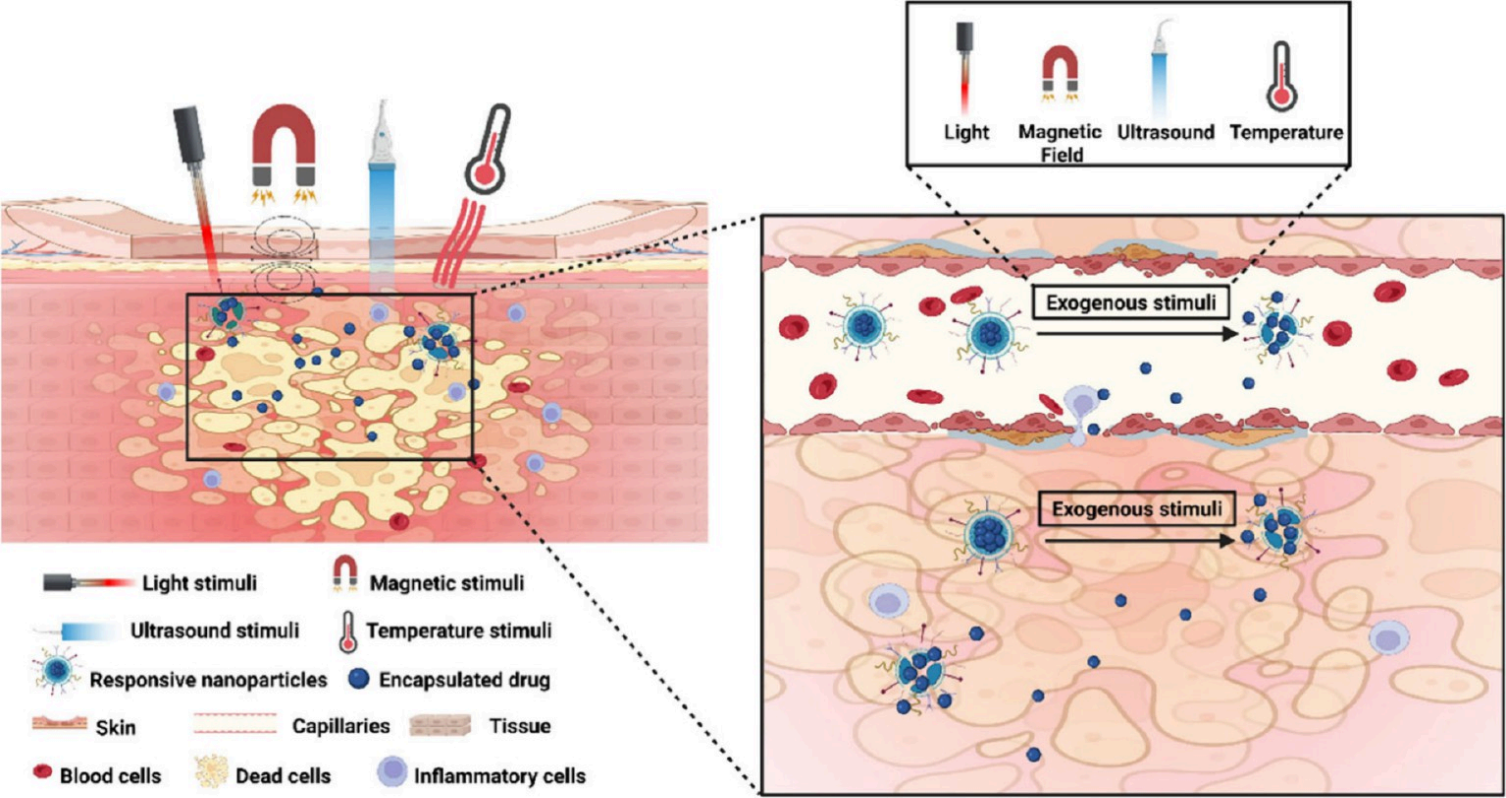
Stimuli-responsive nanoparticles triggered by exogenous stimuli. Following the accumulation of stimuli-responsive nanoparticles (SR-NPs), the penetration of external stimuli, such as light, magnetic fields, ultrasound, and temperature, can control drug release at sites of inflammation. Created with BioRender.com.

Exogenous stimuli, such as light, magnetic fields, ultrasound, and temperature, can induce specific drug release from SR-NPs (Figure 7). Advantages of using an exogenous stimulus include precise control over the site and time of drug release, controllable stimulation time and frequency, sustained drug release, and the ability to overlay multiple stimuli.^18^ Therefore, SR-NPs that react with exogenous stimuli have immense potential in diagnosing and treating inflammatory diseases (Table 2).

**Table 2:** Summary of Stimuli-Responsive Nanoparticles Triggered by Exogenous Stimuli

Exogenous stimulus	Sensitive building block	Nanocarrier	Result	Therapeutic application	ref
**Light**	Polydopamine	Hydrogel	Promoted angiogenesis and mitigated inflammation	Wound	(126)
**Light**	Nd^3+^	Upconversion nanoparticles	Enhanced angiogenesis, reduced infarction and inflammatory responses, and induced repair of brain tissues	Stroke	(128)
**Light**	Yb^3+^	Upconversion nanoparticles	Biocompatible and penetrates deep tissue; used for imaging and therapy	Neuronal diseases	(129)
**Magnetic**	Iron oxide	Superparamagnetic iron oxide particles	Tracked inflammatory cells to sites of infection and inflammation in an *in vivo* murine model	Infection-induced inflammation	(142)
**Magnetic**	Superparamagnetic iron oxide nanoparticles	Hydrogel	Improved the targeted delivery capabilities. The results corroborate the formulation’s efficacy, improving the treatment	Osteoarthritis	(143)
**Ultrasound**	Microbubbles	Diclofenac	Increased skin permeability and enhanced diclofenac delivery to inhibit inflammation	Osteoarthritis	(150)
**Ultrasound**	Nanozyme	Hydrogel	Reduced inflammation, relieved hypoxia, lowered blood glucose, promoted angiogenesis, and eliminated pathogenic bacteria, thus accelerating diabetic wound healing	Diabetic wounds	(151)
**Ultrasound**	Perfluoropentane	Perfluoropentane–hematoporphyrin monomethyl ether@poly(lactic-*co*-glycolic acid)/manganese ferrite	Sonodynamic therapy inhibited plaque neovascularization by inducing mitochondrial-caspase-mediated apoptosis in neovascular endothelial cells and rapidly reduced plaque inflammation	Atherosclerosis	(153)
**Temperature**	Pluronic F127	Chitosan oligosaccharide conjugated pluronic F127 grafting carboxyl group nanoparticle	Reduced cyclooxygenase-2 expression in the serum and synovial membrane of treated rats and induced inflammation	Osteoarthritis	(157)
**Temperature**	*N*-isopropylacrylamide-*co*-butyl methyl-acrylate	P(N -isopropylacrylamide-*co*-butyl methacrylate) nanogel	Embolic agent-induced reduction in inflammation	Vascular occlusion	(158)

#### 3.2.1 Light-Responsive Nanoparticles

Light irradiation represents a widely recognized means of stimulating or triggering drug release from SR-NPs.^120^ Light sources can be turned on or off instantaneously and directed to specific subcellular locations with tunable wavelengths and intensities. Near-infrared (NIR) light with a wavelength of 700–1000 nm exhibits robust tissue penetration and induces minimal photodamage; therefore, NIR-responsive nanomaterials designed and synthesized for bioimaging, theranostics, and drug delivery hold immense potential in inflammation-related diseases.^121,122^ While the use of light as a trigger can be applied to a multitude of approaches (including energy conversion for photoablation^123^), the main types of tailored nanoparticles are NIR-responsive photothermal absorbers and lanthanide-doped upconversion nanoparticles (UCNPs).

**Figure 8:**
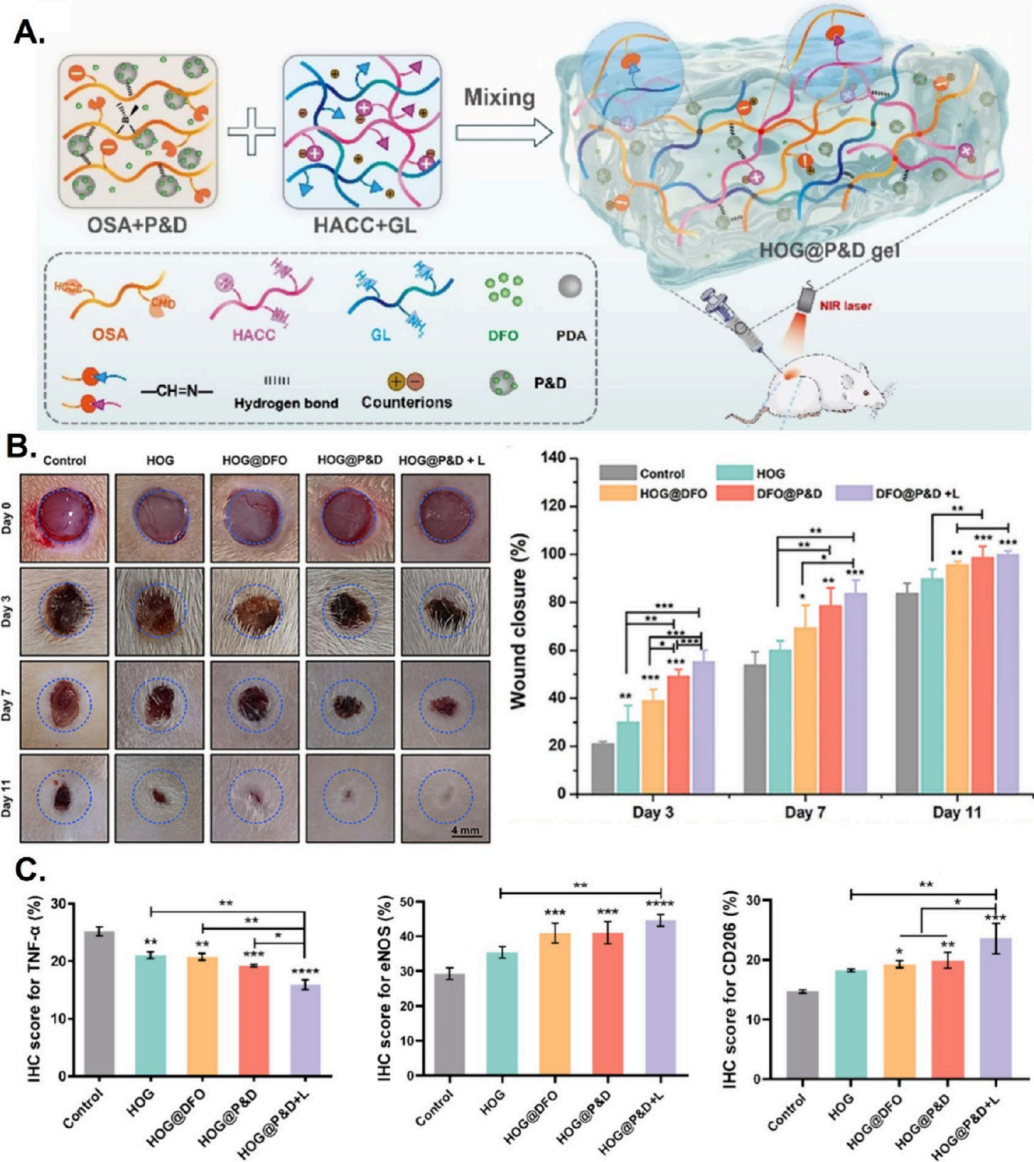
Full-thickness wound healing promotion by HOG@P&D light-responsive hydrogels. (A) Formation mechanism of the injectable extracellular matrix (ECM)-mimicking hydrogel, HOG@P&D. (B) Representative photographs of the wounds, and graph representation of the wound closure of HACC/OSA/GL (HOG), HOG@DFO, HOG@P&D, HOG@P&D + L (NIR laser) groups during the healing process; controls are group were treated with normal saline. (C) TNF-α and eNOS immunohistochemical staining score of various groups on day 7, and CD206 immunohistochemical staining score of various groups on day 3. Reproduced with permission from ref (126). Copyright 2025 Elsevier BV.

NIR-responsive photothermal absorbers trigger a localized increase in temperature,^124,125^ and Wang et al.^126^ developed an injectable extracellular matrix (ECM)-mimicking hydrogel for wound healing. Taking advantage of the Schiff base and hydrogen bonds among a N-2-hydroxypropyl trimethylammonium chloride chitosan (HACC), oxidized alginate (OSA), gelatin (G), deferoxamine (DFO), and polydopamine (P&D) nanoparticles, the authors engineered a hydrogel (HOG@P&D) with the capacity to respond to NIR irradiation, converting laser energy into heat to trigger an on-demand release of DFO, thereby effectively enhancing angiogenesis. Importantly, this hydrogel showed antibacterial and antioxidant properties, promoted angiogenesis, and reduced inflammation by decreasing TNF-α and eNOS. Results showed that on Day 7 the hydrogel promoted full-thickness wound healing (80%) compared to the control (60%) (Figure 8).

Lanthanide-doped UCNPs function as transducers, converting NIR energy into visible light to activate canonical optogenetic opsins, which induce the opening of ion channel pores within the cell membrane to prompt membrane depolarization/hyperpolarization.^127^ Wang et al. employed core–shell neodymium (Nd^3+^)-doped UCNPs to convert 808 nm NIR into tissue-penetrating visible light as a component of an ischemic stroke treatment administered *via* brain stereotactic injection.^128^ This UNCP formed part of a NIR-driven nanophotosynthetic biosystem that drives the cyanobacteria Synechococcus elongatus to produce oxygen, enhance angiogenesis, reduce infarction and inflammatory response, facilitate repair of brain tissues, and protect neurons from ischemic insults to improve stroke outcome. In a related study, Wu et al. achieved the enhanced upconversion of luminescence in dye-sensitized core/active shell UCNPs *via* ytterbium ion (Yb^3+^) doping, allowing the transfer of energy from the dye to the UCNP core.^129^ By loading poly(methyl methacrylate)-based biocompatible implantable systems with dye-sensitized core/active shell UCNPs, the optogenetic neuronal excitation window shifted from 808 to 561 nm, which penetrates deeply in tissues. Unfortunately, UCNPs suffer from significant limitations, including concerns related to the long-term biocompatibility of inorganic UCNPs.^121^

Light stimulation also represents a fascinating regulator of nanozyme function.^130^ Nanozymes - artificial enzymes - exhibit enzyme-like catalytic properties and have intrinsic advantages over natural enzymes, such as low cost, high stability, and the potential for large-scale production.^131^ Moreover, nanozymes display greater multifunctionality and the ability to be modulated when compared to conventional enzyme mimics,^132^ which has allowed the light-mediated control of nanozyme function and their application to biosensing, organic pollutant degradation, DNA-modification, and antibacterial approaches.^133,134^

#### 3.2.2 Magnetic Field-Responsive Nanoparticles

Nanoparticles that respond to magnetic fields play significant roles in applications such as drug delivery, hyperthermia, cell separation, and imagining.^135^ These nanomaterials contain elements (e.g., iron, cobalt, nickel, or manganese) affected by magnetic fields, with physical and chemical properties optimized to improve their utility in biomedicine by adjusting size, shape, structure, and components.^136^

**Figure 9:**
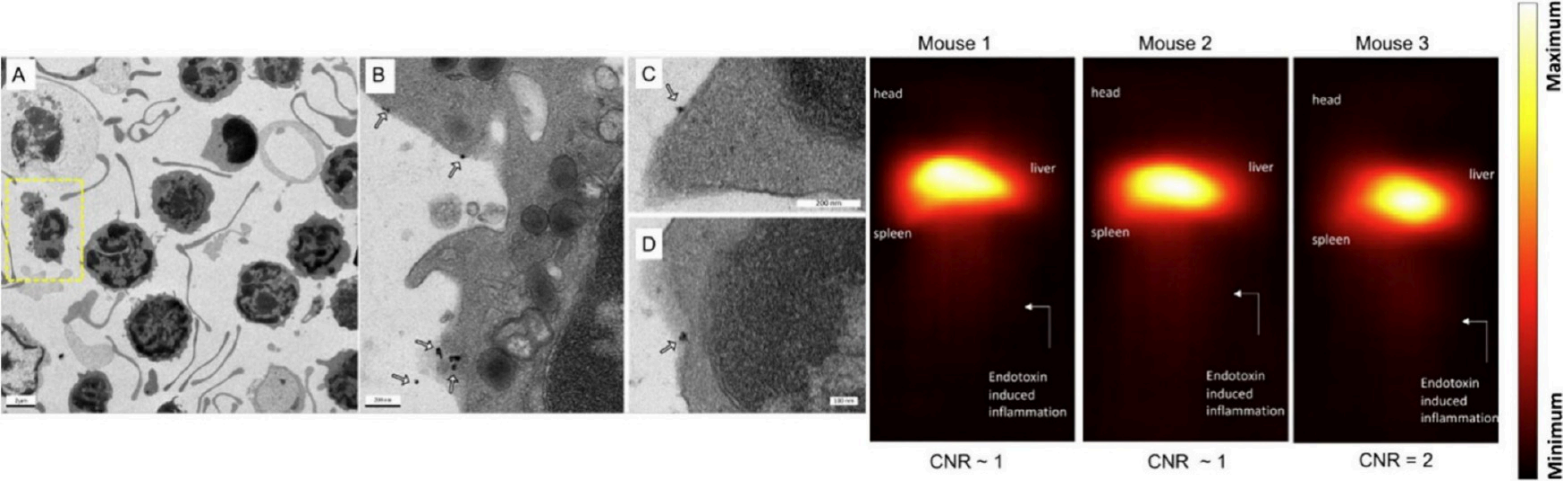
Labeling of immune cells using anti-Ly6G-conjugated superparamagnetic iron oxide particles. (A) Transmission electron microscopy (TEM) images of whole blood samples incubated with anti-Ly6G SPIO. Scale bar = 2 μm. (B and C) Magnified TEM images. Scale bar = 200 nm. (D) Scale bar = 100 nm. The arrows point to the blood-cell-membrane-bound nanoparticles. (E) Images of three animals using VivoTrax as a tracer in a mouse myositis model. At a dose of 5 mg of Fe/kg, the CNR was ∼1–2 at the site of myositis acquired 24 h post-tracer administration (CNR: contrast-to-noise ratio). Reprinted with permission under a Creative Commons CC BY 4.0 from ref (142). Copyright 2021 Nanotheranostics.

Magnetic field-responsive nanoparticles, such as iron oxide nanoparticles (IONPs, which include superparamagnetic iron oxide particles [SPIOs] and ultrasmall superparamagnetic iron oxide particles [USPIOs]), have been widely studied as imaging agents for the diagnosis of inflammatory disease.^137,138^ Ligand-conjugated IONPs have been widely studied as targeted contrast agents for the molecular imaging of atherosclerosis, thrombosis, and myocardial infarction.^139,140^ Merinopoulos et al.^141^ also reported the uptake of USPIOs by the monocytes and macrophages that accumulate at sites of inflammation. Chandrasekharan et al.^142^ employed anti-Ly6G antibody-conjugated SPIOs to selectively tag the neutrophil-specific Ly6G antigen and thereby robustly distinguish sites of LPS-induced myositis, allowing the tracking of these inflammatory cells to sites of infection and inflammation *in vivo* in a murine model (Figure 9). Mushtaq et al.^143^ designed an *in situ* forming hydrogel incorporating flurbiprofen-loaded bilosome and SPIONs to impart magnetic responsiveness, which improved the targeted delivery capabilities. A 27.83% reduction in joint inflammation and an 85% improvement in locomotor activity in osteoarthritic rats treated with this hydrogel was observed with respect to controls.

While SPIOs have been widely applied for various biological and medical purposes, such as *in vivo* imaging, biomolecule detection, and drug delivery,^144^ their broad adoption remains challenging due to their inaccurate spatiotemporal localization in tissues after a certain depth. Maintaining a sufficiently strong magnetic field - as the magnetic field gradient rapidly decreases with distance – represents the main limitation.^135^

#### 3.2.3 Ultrasound-Responsive Nanoparticles

The application of ultrasound-responsive nanoparticles represents a relatively mature and promising medical technological approach in medical imaging and drug delivery,^145,146^ with characteristics such as energy concentration, improved penetration depth, safety, easy operation, and low cost. Ultrasound-responsive nanoparticles release drugs through cavitation, mechanical effects, and localized thermal effects under the influence of ultrasonic waves.^147,148^ Ultrasound can also enhance drug cell penetration by disturbing cell membranes and enable intracytoplasmic drug delivery by perforating the cell membrane through shock waves and microjets generated by inertial cavitation.^148,149^ Notably, ultrasound increases skin permeability and enhances anti-inflammation drug delivery, inhibiting tissue inflammation.^150^

**Figure 10:**
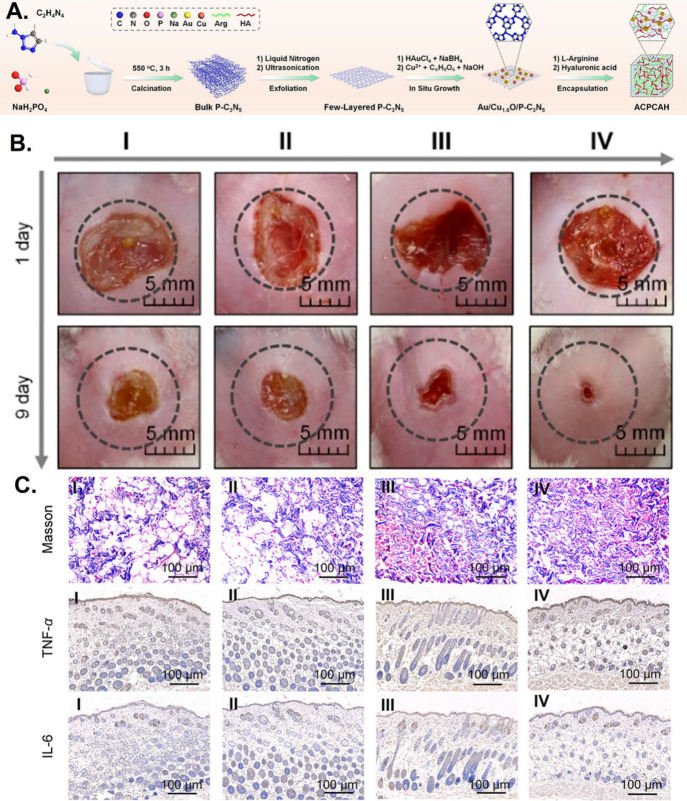
ACPCAH: Ultrasound-augmented multienzyme-like nanozyme hydrogel spray promotes diabetic wound healing. (A) Synthetic route for ACPCAH development. (B) Traces of wound closure within 9 days after different treatments (Control (I), Ultrasound (II), ACPCAH (III), and ACPCAH+Ultrasound (IV)). (C) Masson staining of wounded tissues after different treatments and representative photographs of TNF-α and IL-6 immunohistochemical staining. Reproduced with permission from ref (151). Copyright 2023 American Chemical Society.

Shang et al. developed an ultrasound-augmented multienzyme-like nanozyme hydrogel spray using hyaluronic acid encapsulated l-arginine, ultrasmall gold (Au) nanoparticles, and Cu_1.6_O nanoparticles coloaded with phosphorus-doped graphitic carbon nitride nanosheets (ACPCAH) as a treatment for diabetic wound healing.^151^ This nanozyme hydrogel spray possessed five enzyme-like activities, including superoxide dismutase, catalase, glucose oxidase, peroxidase, and nitric oxide synthase-like activities. Coupling nanozyme-associated catalysis and sonocatalysis under ultrasound stimulation further boosted ACPCAH’s therapeutic efficacy - reducing inflammatory cytokines TNF-α and IL-6 expression, relieving hypoxia, lowering blood glucose levels, enhancing angiogenesis, and eliminating pathogenic bacteria to accelerate diabetic wound healing (Figure 10). In a separate study, Shang et al. encapsulated perfluoropentane (PFP) – which can be vaporized into gas microbubbles using ultrasound^152^ - in PLGA shells, with the resultant nanoparticles converted into microbubbles under low-intensity focused ultrasound irradiation.^153^ They employed this ultrasound-responsive nanoparticle as an ultrasound contrast agent to diagnose cardiovascular plaque disease.

Ultrasound-responsive nanoparticles face challenges related to the difficulty of improving material sensitivity/ultrasound responsivity and in vivo material instability.^154^ At the same time, parameters such as ultrasonic irradiation, concentration, and molecular weight of the therapeutic drug loaded into ultrasound-responsive nanoparticles can significantly affect the release efficiency.^148^

#### 3.2.4 Temperature-Responsive Nanoparticles

Given the elevated temperatures at sites of inflammation compared with healthy tissues, temperature-responsive nanoparticles can be used for the precise and localized delivery of drugs. Said nanoparticles take advantage of the temperature differential described above or external thermal stimulation for drug release.

The aggregation properties of temperature-responsive copolymers can modify nanoparticles to enable thermotaxis.^155^ Temperature-responsive nanoparticles typically contain components that remain stable at body temperature but promote drug release in response to an increase in the surrounding temperature (>10 °C compared to body temperature) due to a change in physical and chemical properties.^17,86,156^

**Figure 11:**
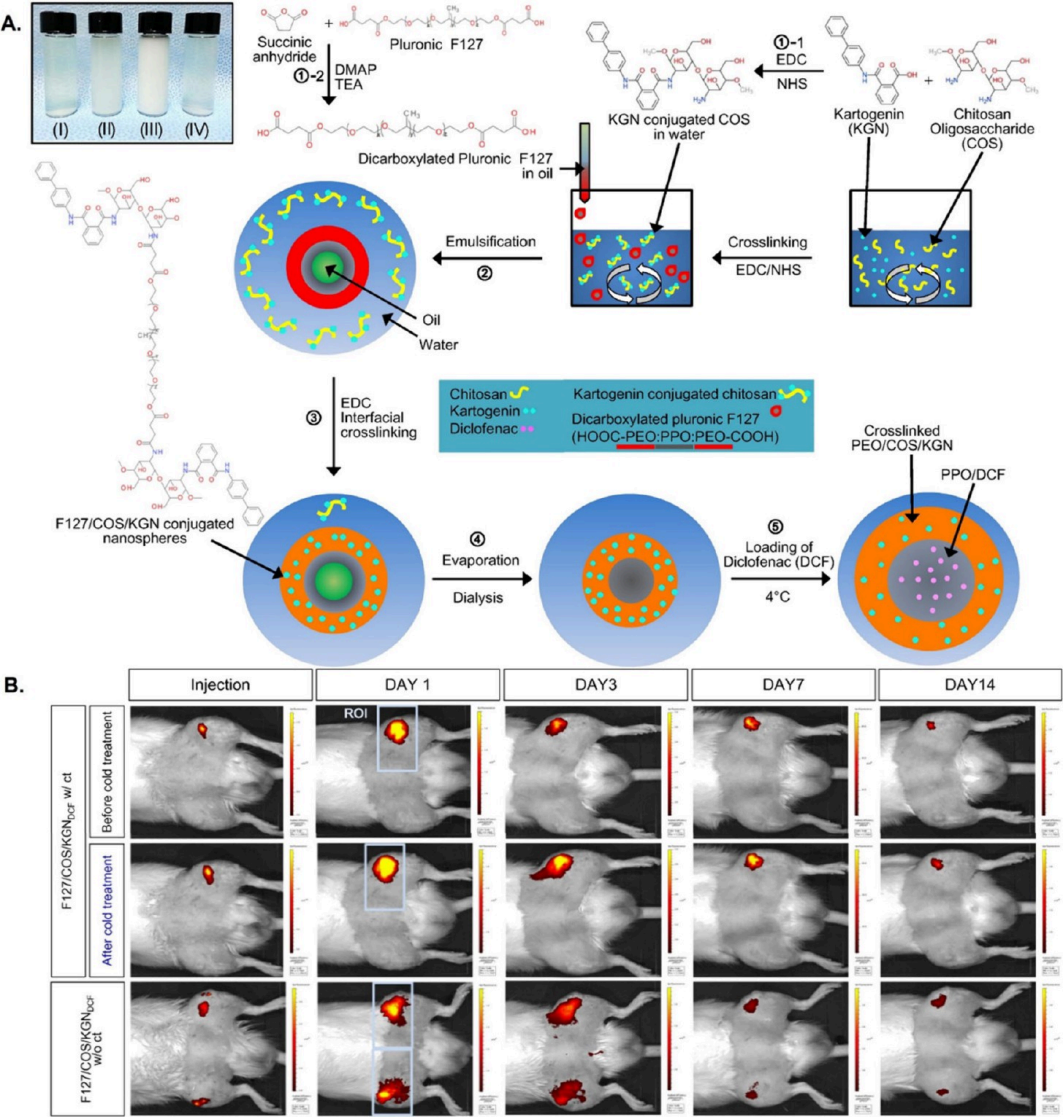
Retention time and thermal responsiveness of F127/COS/KGNDCF nanospheres in osteoarthritic joints. (A) Synthetic scheme used to develop F127/COS/KGNDCF nanospheres. (B) Retention time and thermal responsiveness of F127/COS/KGNDCF nanospheres in osteoarthritic joints. *In vivo* bioluminescence imaging using fluorescent dye-labeled F127/COS/KGNDCF nanospheres at various time points in osteoarthritic rats receiving or not receiving cold treatment. The scale bar range is 0.1–3.5 × 10^–8^ in bioluminescence intensity. Reproduced with permission from ref (157). Copyright 2016 Elsevier.

Kang et al.^157^ synthesized thermoresponsive nanospheres (F127/COS/KGNDCF) comprising an outer layer of cross-linked dicarboxylate pluronic F127 (F127-COOH)/chitosan oligosaccharide (COS)/kartogenin (KGN) and an inner layer of F127-COOH loaded with an anti-inflammation drug, diclofenac (DCF), for osteoarthritis treatment. Increased temperature altered the nanosphere volume *via* F127, which induced DCF release and the subsequent inhibition of LPS-induced inflammation in chondrocytes and macrophages in a rat osteoarthritis model (Figure 11). Additionally, Zhai et al. reported using a temperature-responsive liquid embolic agent, which remains liquid at low temperature and solidifies at body temperature, for angiography.^158^

### 3.3 Dual- and Multiresponsive Nanoparticles

While nanoparticles that respond to a single stimulus ensure the site-specific release of drugs, dual- and multiresponsive nanoparticles that release drugs in response to a combination of differing stimuli can support improved safety and targeting accuracy, higher loading efficiency, and sustained release times and display a better ability to sense slight changes in the microenvironment.^85^ Examples of nanoparticles that respond to dual stimuli include those responding to altered pH and ROS levels,^159−161^ pH and hypoxia,^162^ pH and light,^163^ temperature and pH,^164^ magnetic fields and pH,^165^ light and magnetic fields,^166^ temperature and redox potential,^167^ and redox/pH and temperature.^168,169^ pH- and ROS-responsivity represents the most common combination due to more straightforward chemistry and system tunability.

**Figure 12:**
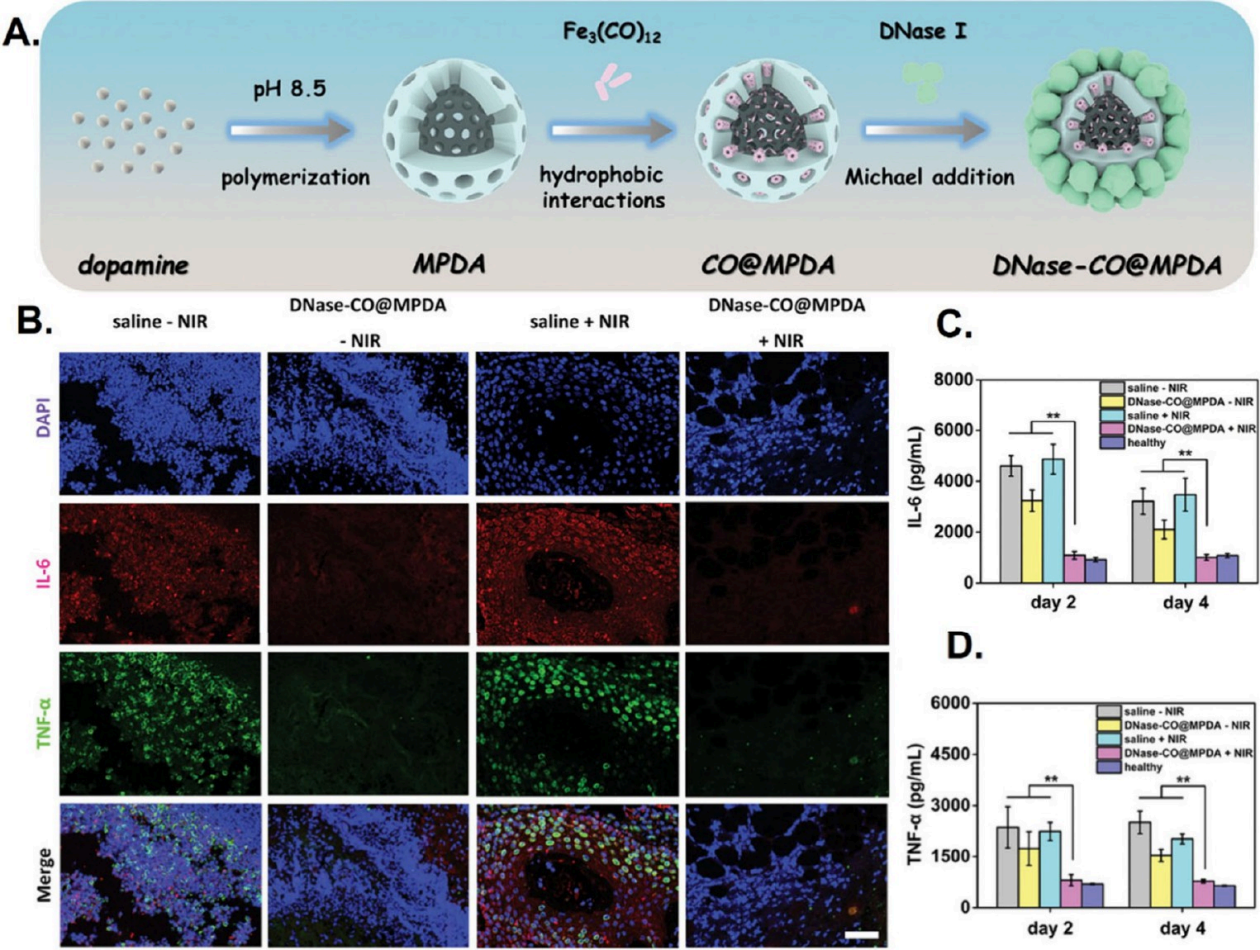
DNase–CO@MPDA nanoparticles: Near infrared light-responsive nanoparticles promote bacterial death and accompanying inflammation. (A) Schematic illustration of the preparation of DNase–CO@MPDA nanoparticles. (B) Corresponding double immunofluorescent staining (IL-6 and TNF-α) of post-treated wounds with/without NIR irradiation. (C and D) Proinflammatory cytokine analysis (IL-6 and TNF-α) in wounds via ELISA. Reproduced with permission from ref (173). Copyright 2021 Wiley VCH.

Taking advantage of the elevated ROS levels and lower pH values associated with the inflammatory environment, Xu et al. grafted 4-(hydroxymethyl) phenylboronic acid pinacol ester (PAPE; ROS-responsive) to a PBAE side chain (pH-responsive) via a succinic anhydride (SA) linker to obtain a pH/ROS dual-responsive nanocarrier (PBAE-SA-PAPE).^170^ They loaded curcumin into nanoparticles and then encapsulated them in a chitosan/alginate hydrogel, which targeted macrophages and supported more rapid curcumin release under conditions of acidic pH and elevated ROS levels, significantly alleviating inflammation in ulcerative colitis mice via the TLR4-MAPK/NF-κB pathway. In a related study, Wang et al.^171^ grafted 3-carboxy-phenylboronic acid to a gelatin backbone and cross-linked with poly(vinyl alcohol) to form a phenylboronic acid–diol ester bond sensitive to pH- and ROS, which released vancomycin and nimesulide in response to inflammation to promote infected wound healing. Lastly, Lee et al.^172^ developed a dual-responsive nanoparticle with high specificity to the inflammatory environment by blending a ROS-responsive dextran-naproxen conjugate with a pH-responsive acetylated dextran polymer. The authors modified the anti-inflammatory COX inhibitor naproxen with a ROS-responsive phenylboronic acid (PBA) linker for conjugation to an acid-sensitive acetylated dextran polymer. The resultant dual-responsive nanoparticle released drugs more rapidly under inflammatory conditions, exerted an anti-inflammatory effect by scavenging ROS, and significantly reduced IL-6 and TNF-α levels.^172^ Yuan et al.^173^ proposed a strategy to tackle bacterial biofilms by encapsulating tri-iron dodecacarbonyl (FeCO) within mesoporous polydopamine (MPDA) nanoparticles before covalently immobilizing deoxyribonuclease I (DNase I) to the nanoparticle surface (DNase–CO@MPDA). DNase I degrades the extracellular DNA present in biofilms to site-specifically interfere with biofilm compactness, while NIR irradiation induces the photothermal activity of FeCO and triggers the release of bactericidal carbon monoxide that permeates impaired biofilms, promotes bacterial death, and decreases the elevated TNF-α and IL-6 expression observed during bacterial infection-associated inflammation after wounds (Figure 12).

Multiresponsive nanoparticles have also been used to diagnose and treat inflammatory diseases, with examples including those that respond to light, temperature, and ROS for bacterial infection wound therapy^174^ and those that respond to light, ROS, and increased enzymatic activity for atherosclerosis theranostics.^175^ These multifunctional nanoparticles are conducive to developing approaches with high sensitivity, robust therapeutic effects, and improved suitability for clinical applications. Displaying responses to multiple stimuli provides unprecedented control over drug delivery and release, resulting in superior anti-inflammatory effects *in vitro* and *in vivo*. While multiresponsive nanoparticles can enable more rapid drug release and enhanced targeting in inflammatory disease treatment, challenges to their translation that remain unaddressed include poor *in vivo* stability and immunocompatibility and elevated toxicity due to increased structural and chemical complexity.^176^

## 4 Therapeutic Applications of Stimuli-Responsive Nanoparticles as Drug Delivery Systems

**Figure 13:**
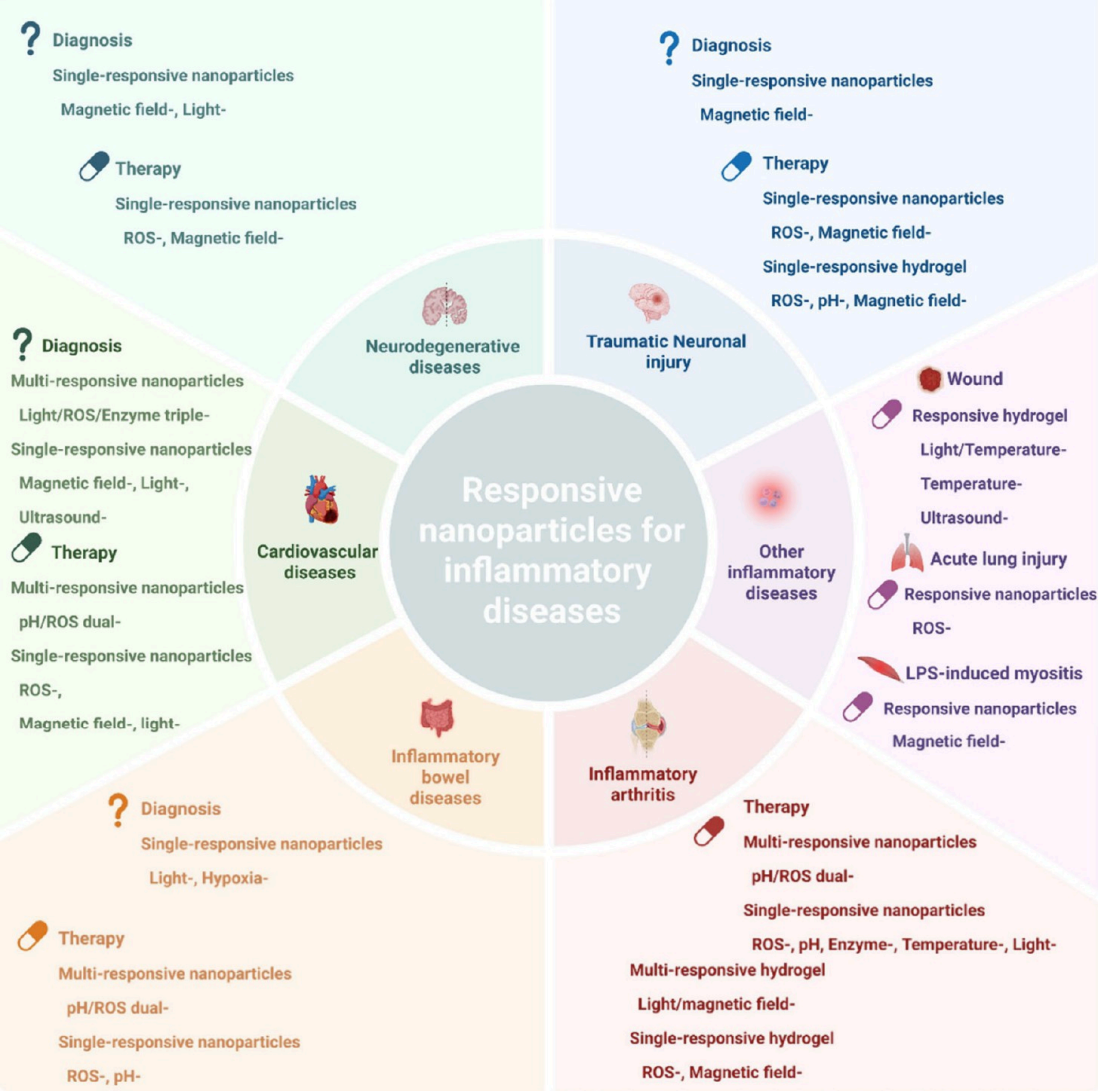
Stimuli-responsive nanoparticles applied to inflammatory disease treatment. Stimuli-responsive nanoparticles are applied to traumatic neuronal injuries, neurodegenerative diseases, cardiovascular diseases, inflammatory arthritis, inflammatory bowel diseases, and other inflammatory diseases. Created with BioRender.com.

This section will illustrate how the mechanisms of the stimulus-responsive NP can be exploited to deliver drugs to treat noncancerous inflammatory diseases (Table 3; Figure 13).

**Table 3:** Summary of Responsive Nanoparticle Applications Applied to Different Inflammatory Diseases

Target diseases	Stimuli	Nanocarrier	Therapeutic agent	Application	Result	ref
**Spinal cord injury**	Reactive oxygen species	Hydrogel	Bone marrow-derived stem cells	Therapy	Reduced scar formation, improved neurogenesis, and enhanced motor function recovery in SCI rats	(96)
**Spinal cord injury**	Temperature	Hydrogel	Astragaloside IV encapsulated in the cavity of apoferritin after an *in situ* biomineralization process involving MnO_2_	Therapy	Effectively ameliorated the oxidative microenvironment post-SCI and inhibited oxidative stress-induced ferroptosis by regulating SIRT1 signaling, thereby promoting neuronal cell migration and repair	(188)
**Spinal cord injury**	pH	Hydrogel	Lysine	Therapy	Improved mitochondrial tricarboxylic acid cycle and fatty acid metabolism, restoring energy supply and facilitating mitochondrial function recovery	(189)
**Spinal cord injury**	Reactive oxygen species	Nanoparticle	IRF5 siRNA	Therapy	Effectively transfected IRF5 siRNA, maintaining high stability and bioactivity, thereby regulating M1-to-M2- like macrophage conversion *in vitro* and *in vivo*. Suppressed excessive inflammation, enhanced neuroprotection, and promoted locomotor restoration after SCI	(192)
**Traumatic brain injury**	Reactive oxygen species/enzymatic activity	Hydrogel	Curcumin	Therapy	Potent anti-inflammatory effects promoted nerve regeneration after TBI	(193)
**Traumatic brain injury**	Reactive oxygen species	Nanoparticle	Nimodipine	Therapy	Inhibited Ca^2+^ influx in neurons and scavenged ROS in the TBI microenvironment to prevent injury-associated secondary injury	(187)
**Epilepsy**	Light	Nanoparticle	Asante Potassium Green-2 tetramethylammonium salt (K-indicators)	Diagnosis	Tissue-penetrating near-infrared emission-based K nanosensors allowed the precise detection of epileptic foci in whole-brain imaging, thereby facilitating the diagnosis and therapy of epilepsy and decreasing the need for surgery	(204)
**Alzheimer’s disease**	Magnetic field	Nanoparticle	α-Synuclein paired antibody	Therapy	Sensor applied to the direct analysis of α-synuclein in diluted serum samples	(206)
**Alzheimer’s disease**	Reactive oxygen species	Nanoparticle	Metal chelator CQ	Therapy	MSN-CQ-AuNPs inhibited Cu^2+^-induced Aβ_40_ aggregation and protected PC12 cells from cell membrane disruption, microtubular defects, and ROS-mediated apoptosis induced by Aβ_40_-Cu^2+^ complexes. The controlled release of CQ from MSN-CQ-AuNPs overcame limitations arising from the nonselective action of CQ.	(209)
**Atherosclerosis**	MRI/Light/ultrasound	Nanoparticle	Manganese ferrite, hematoporphyrin monomethyl ether, and perfluoropentane	Diagnosis/Therapy	With excellent MRI/photoacoustic/ultrasound imaging ability, the distribution of PHPMR nanoparticles in plaque can be observed in real-time. Induced apoptosis in neovessel endothelial cells and ameliorated hypoxia in advanced plaques reduced the density of neovessels, subsequently inhibiting intraplaque hemorrhage and inflammation, and eventually stabilizing the plaque.	(153)
**Atherosclerosis**	Light/MRI	Nanoparticle	Macrophage receptor with collagenous structure, MARCO	Diagnosis	Binding affinity to M1-like macrophages could be applied for noninvasive dual MRI and optical imaging of M1-like macrophage behavior in vulnerable atherosclerotic plaques	(216)
**Atherosclerosis**	Light/MRI	Nanoparticle	Profilin-1	Diagnosis	Used as molecular imaging probes to visualize atherosclerotic plaque in apoE^–/–^ mice *in vivo* through near-infrared fluorescence and MRI	(218)
**Cardiovascular disease**	pH/Reactive oxygen species	Nanoparticle	pH-sensitive (ACD) and oxidation-responsive materials (OCD) (AOCD)	Therapy	In response to low pH or an elevated level of H_2_O_2_, rapamycin/AOCD nanoparticles release rapamycin	(159)
**Atherosclerosis**	Reactive oxygen species	Nanoparticle	CD47 antigen receptor	Therapy	Promoted metabolic reprogramming and improved the LXR signaling vital in maintaining cholesterol homeostasis and reducing inflammation	(220)
**Arthritis**	Enzymatic activity	Nanoparticle	MMP-2/9 sensitive peptide	Therapy	Reduced clinical arthritis index and serum cytokines in adjuvant-induced arthritis	(113)
**Arthritis**	pH	Nanoparticle	Triptolide	Therapy	Decreased infiltration of CD3+ T cells and F4/80+ macrophages and reduced TNF-α, IL-6, and IL-1β expression in inflamed lesions in a collagen-induced arthritis mouse model	(89)
**Arthritis**	Temperature	Nanoparticle	Elastin-like polypeptides	Therapy	Spontaneously aggregated upon injection into the knee joint, extending the joint half-life and sustaining the release of the free peptide in the joint fluid	(231)
**Osteoarthritis**	Reactive oxygen species	Nanoparticle	Dexamethasone and CDMP-1	Therapy	Significantly reduced TNF-α, IL-1β, and type II collagen expression levels, inhibited activated macrophages, and reduced inflammatory responses caused by LPS	(104)
**Osteoarthritis**	Magnetic	Hydrogel	Flurbiprofen-loaded bilosomes	Therapy	Improved the targeted delivery capabilities. The results corroborate the formulation’s efficacy, improving the treatment	(143)
**Osteoarthritis**	Temperature	Nanoparticle	Kartogenin and diclofenac	Therapy	Suppressed osteoarthritis progression in treated rats and reduced cyclooxygenase-2 expression in the serum and synovial membrane	(157)
**Rheumatoid arthritis**	Light	Nanoparticle	Methotrexate	Therapy	Ameliorated clinical signs of arthritis, suppressed serum levels of pro-inflammatory cytokines and anti-CII IgG, reduced inflammation, and prevented bone erosion in the joints	(232)
**Rheumatoid arthritis**	Magnetic field	Hydrogel	Flurbiprofen	Therapy	Enhanced entrapment efficiency and targeted delivery capabilities, reduced joint inflammation, and improved locomotor activity in osteoarthritic rats	(143)
**Rheumatoid arthritis**	Ultrasound	Hydrogel	Diclofenac	Therapy	Ultrasound combined with microbubbles increased skin permeability and enhanced the delivery of diclofenac sodium gel, inhibiting inflammation of the tissues surrounding the arthritic ankle	(150)
**Inflammatory bowel disease**	Light	Nanoparticle	N,N-diphenylnaphthalen-1-amine-(benzo[1,2-c:4,5-c′]bis[1,2,5]-thiadiazole)	Diagnosis	Accurately traced inflammatory lesions, monitored inflammation severity, and detected responses to drug intervention in IBD mouse models	(240)
**Inflammatory bowel disease**	Reactive oxygen species	Nanoparticle	Genistein	Therapy	Attenuated the infiltration of inflammatory cells, promoted autophagy of intestinal epithelial cells, inhibited the secretion of IL-1β and TNF-α, modulated the gut microbiota, and alleviated colitis	(241)
**Inflammatory bowel disease**	Reactive oxygen species	Nanoparticle	TotalROX	Diagnosis	Measured ROS produced by cells under inflammatory conditions, evaluated the degree of colitis in animal models, and provided an approach for diagnosing inflammation in IBD with fluorescence-guided colonoscopy	(242)
**Inflammatory bowel disease**	pH	Nanoparticle	Tacrolimus	Therapy	Facilitated a high drug concentration within the sites of intestinal inflammation and improved the drug levels in target tissues, thus avoiding systemic side effects and improving efficacy	(88)
**Inflammatory bowel disease**	pH/Reactive oxygen species	Nanoparticle	Quercetin and mesalazine	Therapy	Accumulated in intestinal inflammation sites and displayed better therapeutic efficacy than the free drugs in a colitis model	(244)

### 4.1 Traumatic Neuronal Injuries

Primary neuronal injuries include traumatic brain injury (TBI) and traumatic SCI, while secondary neuronal injuries, including the neuroinflammation that follows primary neuronal injuries, contribute to metabolic and cellular dysfunction at the injury site and periphery.^177^ Secondary injuries and especially chronic inflammation significantly impact injury severity;^178^ therefore, prompt and effective interventions could reduce mortality.^179,180^ Current treatment strategies include neuroprotective therapies, such as surgical decompression, methylprednisolone to reduce inflammation, and blood pressure augmentation; however, their unsatisfactory therapeutic efficiency does not currently support lesion repair.

USPIOs were employed to assess leukocyte (mainly macrophage) infiltration by magnetic resonance imaging (MRI) to monitor the dynamic inflammatory response to TBI to provide a more accurate and specific description of the inflammatory response as a diagnosis tool for traumatic neuronal injuries.^181^

Cell therapy represents a promising treatment for traumatic neuronal injuries that has been evaluated in Phase I clinical trials (NCT01325103, NCT02482194);^182^ however, the presence of a toxic neuroinflammatory microenvironment at injury sites has limited efficacy.^183^ To address this challenge, researchers have developed strategies using nanoparticles,^184^ exosomes,^185^ scaffolds,^186^ and hydrogels^187^ as carriers to deliver stem cells and anti-inflammatory drugs. ROS-,^96^ temperature-,^188^ and pH-responsive^189^ hydrogels represent an oft-employed means of delivering drugs or stem cells to the relatively limited and defined SCI lesion.^190,191^ ROS-,^96^ temperature-,^188^ and pH-responsive^189^ hydrogels have all been employed for this purpose; however, ROS-responsive nanoparticles and hydrogels are most often used to decrease inflammation after SCI.^187,192,193^

Injuries to the central nervous system may lead to severe locomotor disabilities, which affect the patient’s social life. Improving or reestablishing an adequate functional state of neurons can promote locomotor recovery. Physical stimulation and smart piezoelectric nanobiomaterials can promote neuronal regrowth to treat injury.^194^ Such nanobiomaterials have succeeded in *in vitro* and *in vivo* experiment setups, yet there seems to be a need to explore the clinical translation of outcomes.

### 4.2 Neurodegenerative Disease

Neuronal damage is a pathological hallmark of neurodegenerative diseases, including Parkinson’s disease (PD), Huntington’s disease, Alzheimer’s disease (AD), multiple sclerosis (MS), and amyotrophic lateral sclerosis (ALS), and a significant cause of morbidity and disability.^195^ Neurodegenerative diseases are frequently associated with chronic activation of an innate immune response in the central nervous system;^27^ however, we still lack safe and effective treatments to control unregulated inflammatory processes in the brain.^196^ To date, the treatment of neurodegenerative diseases has focused on counteracting oxidative and inflammatory stress and inhibiting apoptosis, although we have yet to optimize therapeutic effects. Current studies focus on gene therapy, microbiota-targeted therapies, neural stem cell transplantation, mesenchymal stem cell-derived exosomes, and nanobased drug delivery systems.^197−201^ Bioresponsive nanomedicine can be combined with these approaches to open the blood-brain barrier to support drug uptake in the brain^202^ to reduce neural apoptosis and inflammation.^196^ Crossing the blood-brain barrier and maintaining a sufficient drug concentration within lesions to induce a therapeutic effect represent significant challenges in treating neurodegenerative diseases.

Developing sensitive, cost-effective, and noninvasive diagnostic tools for neurodegenerative diseases remains challenging. Magnetic field- and light-responsive nanoparticles have been developed for the early stage diagnosis and monitoring of brain activity in neurodegenerative diseases, providing the possibility of early treatment.^203−205^ Mandala et al. employed IONPs paired with antibodies for the selective detection of α-Synuclein in patient serum samples to diagnose PD,^206^ while Oyarzún et al. employed plasmonic nanoparticles as optical sensing probes for AD detection.^205^ As counteracting oxidative and inflammatory stress is the important key to therapeutic, ROS-responsive nanoparticles represent a common means of targeting neurological lesions, which suffer from a characteristic high level of oxidative stress.^207−209^

Effective treatments to halt neurodegenerative disease progression remain elusive due to several factors, including challenges in drug delivery across physiological barriers like the blood-brain barrier and patient compliance issues leading to treatment discontinuation. Overcoming these issues will require further research to develop noninvasive treatment strategies, improve drug-loading capacities, provide controlled drug release, and increase bioavailability and biocompatibility.^196^

### 4.3 Cardiovascular Diseases

CVDs pathogenesis closely relates to vascular inflammation; for example, atherosclerosis is an inflammatory disease of the artery walls and represents the leading underlying cause of CVDs.^210^ Current CVDs treatments involve both surgery and drug treatments, although long-term treatment with antiplatelet aggregation, lipid-lowering, and antihypertensive drugs can produce adverse reactions, such as gastrointestinal ulcers, arrhythmias, and hypotension. Strategies have been proposed to prevent and treat CVDs by modulating different molecular (e.g., macrophage receptors with collagenous structure and Profilin-1) and cellular processes involved in the inflammatory response.^211,212^

Vascular inflammation causes elevated levels of cholesterol and lipids to accumulate in specific sites of the arteries, inducing an irregular inner surface of the blood vessels. The accumulation and deposition of lipids and cholesterol form atherosclerotic plaques within the artery wall, narrowing the blood vessels and inducing CVDs. Vulnerable plaques display high levels of macrophages and possess a thin fibrous cap and large necrotic lipidic cores.^213^ Light- and magnetic field-responsive nanoparticles^214,215^ can be employed as MRI, computed tomography, near-infrared, and fluorescence imaging agents to detect atherosclerotic plaques.^216−218^ Zones in the artery more prone to CVDs are identified using wall shear stress and oscillatory shear index parameters.^219^ In addition, pH/ROS responsive nanoparticles have also been designed for the treatment of CVDs, given the low pH and high ROS characteristics of vascular inflammation in CVDs caused by oxidative stress damage observed during cardiovascular and other vascular surgery.^159,220^

Currently, stimuli-responsive nanoparticles applied to CVDs are mainly used in diagnosis and ROS removal. The cardiac regeneration potential related nanoparticles application is an exciting strategy for treating CVDs.^221^ The regenerative potential of the heart is extremely limited in adults; however, the myocardial delivery of stem cells or proteins to stimulate cardiac stem cells may lead to the new formation of myocytes and coronary vessels and improve cardiac function after ischemia.^222^ Therefore, stimuli-responsive nanoparticles that deliver stem cells may represent a promising treatment for CVDs.

### 4.4 Inflammatory Arthritis

The term inflammatory arthritis encompasses rheumatoid arthritis and osteoarthritis.^223^ Rheumatoid arthritis is often treated with immunosuppressive agents, such as NSAIDs and corticosteroids, dietary interventions, exercise, and physical therapy. Severe cases require joint replacement to reduce joint pain and swelling, prevent deformities, maintain a certain quality of life, and control extra-articular manifestations.^224^ The immunosuppressive agents employed cause liver damage, gastrointestinal ulcers, teratogenesis, and hair loss; moreover, the bioavailability and efficacy of antiarthritic drugs remain low due to low cell permeability, poor water solubility, random distribution *in vivo*, unfavorable pharmacokinetics, and uncontrolled drug degradation before reaching target sites.^17^ Therefore, designing nanoparticles that respond to factors within the arthritis pathological environment has attracted increasing attention.^148^ Pathological changes of arthritis, including cartilage degeneration, inflammatory arthritis-related synovitis, and subchondral bone restoration, are predominantly induced by changes in microenvironmental factors (such as abnormal levels of degradative enzymes, the disorder of the intracellular redox system, and increased acidic environment).

Due to the expression of enzymes such as MMPs, cysteine proteases, and hyaluronidases during the progression of arthritis, enzyme-responsive nanoparticles have been employed for precise drug release in inflamed joints.^225−227^ At the same time, inflamed lesions give rise to a weakly acidic and ROS-enriched microenvironment; hence, pH-responsive and ROS-responsive nanoparticles have also been used to deliver and release drugs within affected sites,^228^ and ROS-responsive nanoparticles can eliminate the oxidative substances in the diseased part, providing protection and treatment.^229,230^

The superficial location of joints allows the straightforward application of external stimuli; as such, light-responsive and temperature-responsive nanoparticles have been applied to treat joint inflammation.^157,231,232^ Magnetic field-, ultrasound-, and light/magnetic dual-responsive hydrogels have also been used to treat inflammatory arthritis due to the specific location of the inflammation.^143,150,174^ To develop enhanced SR-NPs for arthritic joints, we must deepen our understanding of arthritis at the molecular level to further develop molecular pharmacology and aggrandize novel materials to encounter target-specific biocompatible drug carriers.

### 4.5 Inflammatory Bowel Disease

The pathogenesis of IBD—a chronic and relapsing inflammatory condition of the gastrointestinal tract (mainly consisting of Crohn’s disease and ulcerative colitis)—remains unclear. IBD may result from aberrant and continuing immune responses to the microbes in our gut and modulated by the genetic susceptibility of the individual;^233^ however, accumulating evidence suggests that oxidative stress, apoptosis, and autophagy play essential roles in the pathogenesis and disease progression.^234,235^ IBD causes significant gastrointestinal symptoms, including abdominal pain, diarrhea, bleeding, anemia, and weight loss, which seriously affect the life quality of the patients.^236−238^ Current IBD treatments involve immunosuppressants, glucocorticoids, and nutritional support; however, these treatments suffer from many limitations, such as severe systemic side effects and poor targeting.^239^

NIR-responsive nanoparticles have been used for fluorescence imaging to accurately trace inflammatory lesions and monitor the severity of inflammation in IBD.^240^ The gastrointestinal tract represents a critical source of ROS, pathogens, and ingested materials, which can cause the production of inflammatory cytokines and other mediators, further leading to oxidative stress. As elevated ROS levels occur in IBD lesions, many studies have focused on ROS-responsive nanoparticles for diagnosis and treatment.^241,242^ Based on the higher colonic pH values (6.4–7.0) than in the stomach (pH 1.5–3.5) and intestine (pH 5.5–6.8), pH-responsive nanoparticles have been used for IBD treatment; however, IBD causes pH values in the lesion to decrease, and so these variations may affect such strategies and impede drug release.^243,244^ We require additional studies to successfully translate this concept into clinical practice due to problems regarding the uptake or binding mechanism and stability during gastrointestinal transit.

## 5 The Challenge of Translating Stimuli-Responsive Nanoparticles to the Clinic

The clinical translation of SR-NPs as drug delivery systems has advanced thanks to successes in ongoing research.^245,246^ Magnetic-field responsive nanoparticles have reached clinical trials in acute coronary syndrome diagnosis (NCT02033447) to evaluate the diagnostic performance of rapid immunomagnetic reduction assays in detecting acute myocardial infarction; however, alternative types of SR-NPs have yet to reach clinical trials (ClinicalTrials.gov).

While SR-NPs decrease systemic adverse effects, improve local drug concentration, maintain drug concentration in the lesion site, and outperform other nanomedicinal formulations with regard to targeting drug delivery and prolonging drug action time because of their hydrophilicity, biocompatibility, and targeted delivery, their broader application faces significant challenges. While certain nanocarriers have shown promising results in specific diseases, they also suffer from drawbacks, such as limited absorption and the need for frequent injections and disadvantages that limit clinical application, such as slow response speed and potential cytotoxicity. SR-NPs may undergo enzymatic degradation or physical entrapment on their way to the desired target site because of the variation of pH range in different diseased or normal organs and cells;^17^ therefore, we must fully understand the pathological features of diseases and manufacture suitable SR-NPs according to the different pathological characteristics of different diseases. The design and development of a therapeutic method for the protection and precisely controlled release of inflammatory diseases can effectively improve the therapeutic effect of inflammatory diseases and reduce side effects. Although SR-NPs have advantages over more traditional treatment options, we know little regarding their accumulation in the natural environment and tissues of living organisms^247^ and the biocompatibility and potential toxicity of nanoparticles;^248,249^ therefore, we must fully understand the toxicity mechanisms of nanoparticles and how they modify the intracellular metabolism of higher organisms to improve the application properties of newly synthesized nanomaterials.^247^ The dynamic differences in endogenous stimuli in healthy and diseased tissues during pathogenesis must be understood, as these parameters impact SR-NP behavior, drug release kinetics, and efficiency.^208^ The delivery efficiency of current drug carriers, controlled drug release profile, and therapeutic effects of this nanomedicine in humans still need to be more fully explored. Importantly, the physicochemical and biological characteristics of any new nanomedicine influence the pharmacokinetics,^250^ and thus its therapeutic and side effects. This is even more critical for SR-NPs. Indeed, the linking chemistry impact on the physicochemical properties of nanoparticles, including size, shape, surface chemistry, composition, and biomaterial selection, plays a pivotal role in their safety and toxicity profiles. Also, with immunotherapy as a rising therapeutic strategy, immunotoxicology aspects of nanomedicines are becoming relevant; therefore, studies involving the complement activation and oxidative stress in T lymphocytes, antigen presentation, and stimulation, and the detection of naturally occurring antibodies to PEG must be undertaken in the view of clinical translation.^251^

Indeed, successfully translating SR-NPs into clinical practice requires an adequate number of design approaches. For example, the use of components that have already received one or more designations (e.g., accepted drugs and biomaterials for a certain pathology, linkers used in the antibody-drugs conjugates), have more chances to be accepted.

In this context, the potential risks associated with nanomaterials, particularly concerning human health, must be taken into consideration. The advancements in risk assessment methodologies tailored for nanomaterials have emphasized the importance of 2D and 3D *in vitro* models as well as ex-vivo and *in vivo* toxicological studies involving invertebrate and vertebrate models for the determination of Lethal Dose 50 (LD50), morphological analysis of tissues or organs, and biochemical markers. Moreover, although regulatory authorities across the globe have made efforts to create guidelines for evaluating the safety and potential toxicity of nanomaterials and nanomedicines, several challenges arise due to the complex nature of certain types of biomaterials and nanomedicines, including SR-NPs. Thus, an effort to homogenize the regulatory frameworks is required.

The current understanding of their therapeutic function and the underlying chemical-biological relationship remains preliminary and insufficient to guarantee commercialization. The biocompatibility, endosome safety, and effectiveness of the long-term systemic use of SR-NPs should also be considered. Finally, biological mechanisms underlying the interaction between SR-NPs and the human body remain inadequately understood.^252^ Another limit to clinical translation relates to the cost of production of SR-NPs as one of the most challenging steps in nanomedicine product development is scaling up. This is even more critical considering SR-NPs that involve multiple steps of chemical reactions, followed by purification and nanomedicine preparation. All of these factors limit the application of SR-NPs in clinical practice. Only a few physical SR-NPs have been evaluated at the preclinical level,^253−255^ but they will shortly represent an essential part of the clinician’s armory in addressing diagnosis and treatment. At the same time, we expect additional SR-NPs from the increasing number of research avenues to end up in preclinical evaluations.

## 6 Conclusion and Perspectives

SR-NPs provide a strategy for delivering bioactive compounds to inflammatory diseases, providing high specificity and multiple functions in drug delivery and improving diagnosis and treatment efficacy. A range of SR-NPs has been developed that exhibit better outcomes than nonresponsive formulations. SR-NPs have the potential to be widely applied for diagnosing, probing, sensing, and treating noncancer-related inflammatory diseases.

As a new type of rationally designed treatment, SR-NP-based drug delivery systems have received extensive attention and rapid development toward integrating inflammation diagnosis and treatment. An ideal SR-NP for clinical use must be tailored for specific clinical functions, robustness, target structures, and complexity changes.^256^ Synthetic schemes must be designed according to the stimuli that SR-NPs must incorporate into the structure, keeping manufacturing processes in mind. Forming SR-NPs for clinical use requires functional groups that can be used to attach imaging or targeting groups as well as different types of molecules can be made covalent with built-in breakup points, increasing their *in vivo* stability until target-docking has succeeded. This requires researchers to conduct an in-depth analysis of each structure and compare how formation changes with complexity.^256,257^

Thus, translating SR-NPs to said applications requires additional efforts to increase the scalability, reproducibility, and formation yields. The benefits of SR-NPs will overcome these challenges and drawbacks: more precise control of drug release to pathological sites, thereby minimizing off-target effects in surrounding healthy tissues; temporally and spatially controllable drug release; and precise design of responsive nanoparticles according to specific pathological microenvironments.

## Vocabulary

Nanomedicine: It refers to the application of nanotechnology in healthcare, aiming to enhance the biopharmaceutical properties of therapeutics and optimize their delivery to targeted sites. By harnessing the unique characteristics of various biomaterials at the nanometer scale (10^–9^ m), nanomedicine plays a pivotal role in advancing personalized, targeted, and regenerative medicine.
Stimuli-responsive Linkers: A class of chemical moieties that can be cleaved by specific endogenous or exogenous triggers at desired sites to release therapeutic or diagnostic agents.
Non-Cancer-related Inflammatory Diseases: These are conditions of inflammatory-related sickness that are not classified as cancer. In this review, we focused on traumatic neuronal injury, neurodegenerative and cardiovascular disease, inflammatory bowel disease, and arthritis.
Inflammation: It is a protective process of the human body that engages immune cells, blood vessels, and molecular mediators. Its primary function is to remove the initial cause of cell damage, eliminate damaged cells and tissues, and initiate tissue repair.
Endogenous stimuli: It refers to internal stimuli that arise within the body, including factors like the pH changes, or the upregulation of biological components such as enzymes or ROS. Alterations in the tissue microenvironment caused by the inflammatory condition can be leveraged to design nanomedicines that specifically target the affected tissue.
Exogenous stimuli: These are external factors controlled from outside of the body, such as light exposure or magnetic fields. These stimuli can be precisely regulated and directed to create specific microenvironmental changes at a targeted location.
